# Haplotype Analysis of Genomic Prediction Using Structural and Functional Genomic Information for Seven Human Phenotypes

**DOI:** 10.3389/fgene.2020.588907

**Published:** 2020-11-26

**Authors:** Zuoxiang Liang, Cheng Tan, Dzianis Prakapenka, Li Ma, Yang Da

**Affiliations:** ^1^Department of Animal Science, University of Minnesota, Saint Paul, MN, United States; ^2^National Engineering Research Center for Breeding Swine Industry, South China Agricultural University, Guangzhou, China; ^3^Department of Animal and Avian Sciences, University of Maryland, College Park, MD, United States

**Keywords:** genomic prediction, haplotypes, SNP, coding gene, non-coding gene, ChIP-seq, haplotype epistasis, human phenotypes

## Abstract

Genomic prediction using multi-allelic haplotype models improved the prediction accuracy for all seven human phenotypes, the normality transformed high density lipoproteins, low density lipoproteins, total cholesterol, triglycerides, weight, and the original height and body mass index without normality transformation. Eight SNP sets with 40,941-380,705 SNPs were evaluated. The increase in prediction accuracy due to haplotypes was 1.86-8.12%. Haplotypes using fixed chromosome distances had the best prediction accuracy for four phenotypes, fixed number of SNPs for two phenotypes, and gene-based haplotypes for high density lipoproteins and height (tied for best). Haplotypes of coding genes were more accurate than haplotypes of all autosome genes that included both coding and noncoding genes for triglycerides and weight, and nearly the same as haplotypes of all autosome genes for the other phenotypes. Haplotypes of noncoding genes (mostly lncRNAs) only improved the prediction accuracy over the SNP models for high density lipoproteins, total cholesterol, and height. ChIP-seq haplotypes had better prediction accuracy than gene-based haplotypes for total cholesterol, body mass index and low density lipoproteins. The accuracy of ChIP-seq haplotypes was most striking for low density lipoproteins, where all four haplotype models with ChIP-seq haplotypes had similarly high prediction accuracy over the best prediction model with gene-based haplotypes. Haplotype epistasis was shown to be the reason for the increased accuracy due to haplotypes. Low density lipoproteins had the largest haplotype epistasis heritability that explained 14.70% of the phenotypic variance and was 31.27% of the SNP additive heritability, and the largest increase in prediction accuracy relative to the best SNP model (8.12%). Relative to the SNP additive heritability of the same regions, noncoding genes had the highest haplotype epistasis heritability, followed by coding genes and ChIP-seq for the seven phenotypes. SNP and haplotype heritability profiles showed that the integration of SNP and haplotype additive values compensated the weakness of haplotypes in estimating SNP heritabilities for four phenotypes, whereas models with haplotype additive values fully accounted for SNP additive values for three phenotypes. These results showed that haplotype analysis can be a method to utilize functional and structural genomic information to improve the accuracy of genomic prediction.

## Introduction

Genomic selection using genome-wide single nucleotide polymorphism (SNP) markers has been widely used in livestock and crop species (Meuwissen et al., 2016; Crossa et al., 2017), and genomic prediction has been applied to the prediction of human phenotypes (Maier et al., 2018; Lello et al., 2019). However, most prediction models were SNP models fitting each SNP as a locus in the mixed model without requiring information of SNP locations or functions. In contrast, haplotype analysis has the potential to use structural and function genomic information for more accurate genomic prediction (Da, 2015). A number of studies on haplotype genomic prediction have been reported (Calus et al., 2008; Villumsen et al., 2009; Boichard et al., 2012; Cuyabano et al., 2015; Hess et al., 2017; Jónás et al., 2017; Jiang et al., 2018; Jan et al., 2019; Sallam et al., 2020; Won et al., 2020). Methods used in these studies to define haplotype blocks for genomic prediction include a fixed number of SNPs per haplotype block (Calus et al., 2008; Villumsen et al., 2009; Jiang et al., 2018; Sallam et al., 2020; Won et al., 2020), fixed block length (Hess et al., 2017; Won et al., 2020), or linkage disequilibrium (LD) blocks (Boichard et al., 2012; Cuyabano et al., 2015; Jónás et al., 2017; Jan et al., 2019; Won et al., 2020). These haplotype studies had mixed results ranging from decreases to substantial increases in prediction accuracy due to haplotypes relative to SNP models, but the reasons for the successes and failures of haplotype genomic prediction were unknown.

To understand the performance of haplotype genomic prediction, an empirical hypothesis postulates that a haplotype additive value is the sum of all SNP additive values and an epistasis value within the haplotype plus a potential haplotype loss (Da et al., 2016). Under this hypothesis, haplotype epistasis is responsible for the successes, and haplotype loss for the failures in haplotype genomic prediction. However, estimates of haplotype epistasis were unavailable and the reason for haplotype loss was unknown. A multi-allelic haplotype method for genomic prediction and estimation (Da, 2015) and a computing pipeline named GVCHAP implementing this method (Prakapenka et al., 2020) are helpful for estimating haplotype epistasis heritability and for investigating the reason of haplotype loss. The multi-allelic method is derived from the quantitative genetics theory of genetic partition of genotypic values. An advantage of this method is the readily available quantitative genetics interpretations of SNP additive and dominance effects, values and variances; haplotype additive and dominance effects, values and variances; and SNP and haplotype additive and dominance heritabilities. The SNP and haplotype heritability estimates can be used to assess the contribution of haplotype epistasis to the phenotypic variance, and to assess the relationship between haplotype epistasis heritability and the increase in prediction accuracy due to haplotypes. The multi-allelic haplotype method has a unique feature for estimating the heritability of each SNP and each haplotype block. The comparison of the profiles of such SNP and haplotype heritability estimates may provide an understanding about the nature of haplotype loss. The GVCHAP computing pipeline has a main program calculating genomic best linear unbiased prediction (GBLUP) of genetic values and genomic restricted maximum likelihood (GREML) estimation of variance components and heritabilities with a computing strategy that greatly reduces the computing time for validation studies and multiple traits, and has a set of utility programs from data preparation to summary of results. This computing pipeline provided necessary and efficient computing tools to investigate numerous haplotype prediction models.

Applying these methods and tools to the Framingham Heart Study data, we aimed at achieving the following objectives for seven human phenotypes. The first objective was to find the best haplotype prediction model for each phenotype by evaluating numerous haplotype models based on structural and functional genomic information. Structural genomic information included fixed chromosome distance and fixed number of SNPs per haplotype block. Functional genomic information included coding genes, noncoding genes, autosome genes that included both coding and noncoding genes, and ChIP-seq data. A major question to answer was whether functional genomic information was relevant to haplotype genomic prediction and whether functional genomic information alone had the best prediction model for any phenotype. For each type of structural and functional genomic information and for each haplotype block size, four haplotype models were evaluated: haplotype additive values only, haplotype and SNP additive values, haplotype additive and SNP dominance values, and haplotype and SNP additive values and SNP dominance values. The observed prediction accuracies of these haplotype models from ten-fold cross validations were compared with those of the best SNP model either with SNP additive values only or with SNP additive and dominance values. The second objective was to assess the contribution of haplotype epistasis to the phenotypic variance, and the relationship between haplotype epistasis heritability and the increase in prediction accuracy due to haplotypes for the best haplotype prediction models. The third objective was to evaluate the performance of different types of functional genomic information for haplotype genomic prediction. Finally, we evaluated the effects of different SNP densities on the accuracy of haplotype genomic prediction to provide indications whether medium SNP densities such as those used in animal genomic prediction might be suitable for haplotype genomic prediction, and to assess the performance of various SNP densities for gene-based haplotype analysis.

## Materials and Methods

### SNP Data

The Framingham Heart Study (FHS) data (2019 version) had 7565 individuals with genotypes of the 500K SNP panel that had 488,146 autosome SNPs. The SNP coordinates were converted to those of GRCh38.p13 using human dbSNP^1^, and 486,356 SNPs had known chromosome positions on GRCh38.p13. From these 486,356 autosome SNPs, two high density sets of SNP data were analyzed for prediction accuracy of haplotype models: the 380K set of 380,705 SNP markers with minor allele frequencies (MAF) of 0.05, and the 320K set of 327,430 SNPs with MAF of 0.10. Most results in this article were derived from these two high-density SNP sets. The 320K SNP set was designed to serve two purposes, to reduce the number of rare haplotypes through the removal of more SNPs with low frequencies than in the 380K set, and to determine the effect of reduced SNP density on haplotype prediction accuracy. To investigate the minimal SNP density required for haplotype genomic prediction, three medium density SNP sets of 42K, 63K and 76K were selected from the 380K and three medium density SNP sets of 41K, 65K and 82K were selected from 320K SNP sets (Supplementary Table 1).

### Phenotype Data

The seven phenotypes analyzed in this study were high density lipoproteins (HDL), low density lipoproteins (LDL), total cholesterol (TC), triglycerides (TG), height (HT), weight (WT), and body mass index (BMI), with 3657-7564 observations (Supplementary Table 2). BMI was calculated using the formula BMI = Weight/(Height/100)^2^ (Cacciari et al., 2002). TG was tested in 1967-1974 and 1996-2005 (Supplementary Figure 1), but the tests in 1967-1974 could not be used. The mean value of TG from the 1967-1974 tests was 310.59, 2.60 times as large as the mean of 115.99 for the 1996-2005 tests (Supplementary Table 3). Therefore, only the TG values tested in 1996-2005 were used in this study. HDL and TC each had one outlier: a value of 206 that was 13.31 SD from the mean for HDL; 647 that was 17.47 SD from the mean for TC; and TG had two outliers, 1499 and 1404 that were 16.41 and 17.37 SD from the mean, respectively, where SD = standard deviations. These outliers were removed in the subsequent analyses of genomic prediction and heritability estimation using haplotype models. Six of the seven phenotypes (except HT) had skewed distributions and used the Box-Cox transformation (Box and Cox, 1964) implemented by a R script to achieve normality (Ma et al., 2010) (Supplementary Figures 2, 3). The λ value for HT was 1.03 (≈ 1.00), indicating that a normality transformation of HT was unnecessary. For the SNP models, five of the six phenotypes after normality transformation consistently had better prediction accuracy than using the original phenotypic values, with BMI being the only exception with better prediction accuracy using the original BMI values than using the normality transformed BMI values (Supplementary Table 4). Therefore, BMI and HT used the original phenotypic values, and the other five phenotypes used the normality transformed phenotypic values (Supplementary Figures 2, 3). In addition, as a comparison with the transformed TG that benefitted most from the normality transformation, the original TG was analyzed. To distinguish between the original and transformed phenotypic values, original HT, BMI and TG values will be denoted as HT_O_, BMI_O_ and TG_O_, while abbreviations for the other five phenotypes using normality transformed phenotypic values remain unchanged as HDL, LDL, TC, TG, and WT.

### Construction of Haplotype Blocks

Haplotype blocks were defined using structural and functional genomic information. Each haplotype block was treated as a “locus” and each haplotype within the haplotype block was treated as an “allele” in the analysis using GVCHAP (Prakapenka et al., 2020). Haplotype blocking was based on two types of structural genomic information and four types of functional genomic information.

Structural genomic information included fixed chromosome distances in kilobases (Kb) ranging from 5 to 1000 Kb per haplotype block, fixed numbers of SNPs per haplotype block ranging 2-500 SNPs per block initially, but only results of 2-100 SNPs will be reported because the 300-SNP and 500-SNP blocks had severely reduced accuracies. For the 380K SNP set with MAF of 0.05, the method of fixed chromosome distance had an increasing number of haplotypes per block as the block size increases, averaging 102.06 haplotypes per block for 50 Kb blocks to 8,703.03 per block for 1000 Kb blocks; an increasing average number of SNPs per blocks as block size increases, ranging from 7.92 SNPs for the 50 Kb blocks to 141.26 SNPs for the 1000 Kb blocks; and a decreasing number of blocks ranging from 47,701 blocks for 50 Kb blocks to 2,695 blocks for 1000 Kb blocks as block size increases (Table 1). In contrast, the method of fixed number of SNPs per block had an increasing chromosome distance as the number of SNPs increases, ranging from 7.26 Kb for the 2-SNP blocks to 724.74 Kb for the 100-SNP blocks (Table 2). Haplotype statistics for the 320K SNP set with MF of 0.10 were described in Supplementary Tables 5, 6.

**TABLE 1:** Statistics of haplotype blocks defined by fixed chromosome distance (380K, MAF = 0.05).

Distance (Kb)	5	20	50	100	150	200	250	300	400	500	1,000
Total number of haplotypes	1,123,531	2,385,225	4,868,466	9,601,998	14,280,344	18,262,393	21,315,586	23,561,371	26,133,717	26,992,496	23,454,663
Number of blocks	92,993	83,603	47,701	25,774	17,455	13,190	10,596	8,840	6,658	5,339	2,695
Average number of haplotypes per block	12.08	28.53	102.06	372.55	818.12	1,384.56	2,011.66	2,665.31	3,925.16	5,055.72	8,703.03
Minimum SNPs per block	2	2	2	2	2	2	2	2	2	2	2
Maximum SNPs per block	16	29	50	75	92	119	148	164	217	260	451
Average number of SNPs per block	2.71	4.25	7.92	14.75	21.8	28.86	35.93	43.06	57.18	71.3	141.26
Minimum distance in block (Kb)	5	20	50	100	150	200	250	300	400	500	1,000
Maximum distance in block (Kb)	5	20	50	100	150	200	250	300	400	500	1,000
Average distance per block (Kb)	5	20	50	100	150	200	250	300	400	500	1,000

**TABLE 2:** Statistics of haplotype blocks defined by fixed number of SNPs (380K, MAF = 0.05).

Number of SNPs per block	2	5	7	9	12	22	30	50	100
Total number of haplotypes	1,472,716	2,619,431	3,667,005	4,812,953	6,676,664	13,241,867	18,101,920	26,127,332	28,784,087
Number of blocks	190,357	76,151	54,395	42,312	31,734	17,317	12,699	7,624	3,817
Average number of haplotypes/block	7.74	34.4	67.41	113.75	210.39	764.67	1,425.46	3,426.98	7,541.02
Minimum SNPs per block	2	5	7	9	12	22	30	50	100
Maximum SNPs per block	2	5	7	9	12	22	30	50	100
Average number of SNPs per block	2	5	7	9	12	22	30	50	100
Minimum distance in block (Kb)	0.01	0.13	0.55	0.55	2.76	10.99	22.08	50.41	165.19
Maximum distance in block (Kb)	4,548.44	11,019.97	23,467.29	29,732.9	29,754.01	29,774.74	25,970.14	29,898.03	30,138.48
Average distance per block (Kb)	7.26	28.83	43.9	58.8	80.71	153.27	210.87	358.19	724.74

Functional genomic information included all autosome genes, coding genes, noncoding genes, and the protein-DNA binding sites from ChIP-Seq data to be short-named as ‘ChIP-seq sites’ or simply ChIP-seq. Autosome genes included both coding and noncoding genes. The autosome gene boundaries and the classification of coding and noncoding genes were based on the Gene Transfer Format (GTF) files^2^. Protein coding genes were classified as coding genes, and all other genes were classified as noncoding genes. Long noncoding RNAs (lncRNAs) were the majority (84-88%) of the noncoding genes with at least two SNPs per gene for haplotype analysis (Supplementary Table 7). The ChIP-seq data were downloaded from ReMap2020^3^. Haplotypes phasing and imputation used Beagle 5.1 (Browning et al., 2018) with default parameters for forty phasing runs per chromosome. The within-population individuals were used as the reference population for any individual being imputed, i.e., the imputation of any individual used the remaining individuals as the reference population.

Haplotypes using functional genomic information were derived from the 380K and 320K SNP sets. For the 380K SNP set with MAF of 0.05, the number of haplotype blocks was 18,080 for all autosome genes, 12,676 for coding genes, and 10,111 for noncoding genes. The average size of the gene blocks was 95.34 Kb for coding genes, 49.82 Kb for noncoding genes, and 90.13 Kb for all coding and noncoding genes of the autosomes. ChIP-seq had 21,474 blocks with average block size of 75.51 Kb (Table 3). A 2-Kb distance was added to either end of each gene or ChIP-seq site. Based on experience that block sizes exceeding 200 Kb mostly had low prediction accuracy for the 380K data, large genes were split into 150 Kb blocks. The coverage of autosomes by functional haplotype blocks with the 4-Kb extension per block was 50.78% by all autosome genes, 37.66% by coding genes, 15.70% by noncoding genes, and 78.19% ChIP-seq haplotype blocks. For the 320K SNP set with MAF of 0.10, the number of blocks was slightly fewer but average block size was slightly larger than those of the 380K set (Table 3).

**TABLE 3:** Statistics of haplotype blocks defined by gene boundaries and ChIP-seq sites.

	380K, MAF = 0.05				320K, MAF = 0.10			
	
	Autosome genes	Coding genes	Non-coding genes	ChIP-seq	Autosome genes	Coding genes	Non-coding genes	ChIP-seq
Total number of haplotypes	7,419,624	5,571,918	1,946,912	13,368,940	6,350,392	4,776,514	1,655,180	11,496,010
Number of blocks	18,080	12,676	10,111	21,474	17,238	12,168	9,262	20,967
Average number of haplotypes per block	410.38	439.56	192.55	622.56	368.39	392.55	178.71	548.29
Minimum SNPs per block	2	2	2	2	2	2	2	2
Maximum SNPs per block	87	87	64	104	82	82	55	87
Average number of SNPs per block	13.49	13.95	8.56	17.63	12.11	12.44	7.98	15.52
Minimum distance in block (Kb)	1.14	1.14	1.78	4.04	1.14	1.14	1.78	4.04
Maximum distance in block (Kb)	150.0	150.0	150.0	150.0	150.0	150.0	150.0	150.0
Average distance per block (Kb)	90.13	95.34	49.82	116.85	92.65	97.2	52.79	118.15
Autosome coverage (Mb)	1557.23	1158.16	463.33	2423.24	1528.13	1134.03	451.90	2393.44
% of autosomes	48.53	36.08	14.44	75.51	47.62	35.34	14.08	74.59
4-Kb extended coverage (Mb)	1629.55	1208.49	503.77	2509.14	1597.08	1182.70	488.95	2477.31
% of autosomes by 4-Kb extended coverage	50.78	37.66	15.70	78.19	49.77	36.86	15.24	77.20

### Mixed Model for GBLUP and GREML

Genomic best linear unbiased prediction (GBLUP) of genetic values and genomic restricted maximum likelihood (GREML) estimation of variance components and heritabilities were calculated using the GVCHAP program (Prakapenka et al., 2020) that implements a multi-allelic mixed model treating each haplotype block as a “locus” and each haplotype within the haplotype block as an “allele.” The mixed model starts with the quantitative genetics model resulting from genetic partition for SNPs (Da et al., 2014) and for multi-allelic loci (haplotype blocks) (Da, 2015), and implements genomic prediction and variance component estimation using a reparameterized and equivalent model resulting from the use of genomic relationship matrices of SNPs and haplotypes. To avoid repeating a large quantity of notations involving the relationship between the original model and the reparameterized and equivalent model (Da, 2019; Prakapenka et al., 2020), the following description only summarizes the starting quantitative genetics model with SNPs and haplotypes, and the variance-covariance matrices using genomic relationship matrices of the reparametrized and equivalent model. The mixed model with SNP and haplotype effects is:

(1) $$\begin{array}{l} y=Xb+Z(W_{\text{α}}{\text{α}}_{o}+W_{\text{δ}}{\text{δ}}_{o}+W_{\text{αh}}{\text{α}}_{ho})+e\\ \;=Xb+Z(a+d+a_{h})+e \end{array}$$

where **Z** = N × n incidence matrix allocating phenotypic observations to each individual = identity matrix for one observation per individual (N = n), N = number of observations, n = number of individuals, *α*_o_ = m × 1 column vector of SNP additive effects, m = number of SNPs, **W**_α_ = n × m model matrix of *α*_o_, *δ*_o_ = m × 1 column vector of column vector for dominance effects of SNP genotypes, **W**_δ_ = n × m model matrix of *δ*_o_, *α*_h_ = n_αh_×1 column vector of haplotype additive effects, n_αh_ = number of haplotype additive effects, **W**_αh_ = n×n_αh_ model matrix of *α*_h_, **b** = c × 1 column vector of fixed effects, c = number of fixed effects, **X** = N×c model matrix of **b**, **a** = **W**_α_α_o_ = n × 1 SNP genomic additive values, **d** = **W**_δ_**δ**_o_ = n × 1 SNP genomic dominance values, **a**_h_ = **W**_αh_α_oh_ = n × 1 haplotype genomic additive values, and **e** = N × 1 column vector of random residuals. Fixed effects included sex and cholesterol treatment as classification variables, and age, glucose and BMI_O_ as covariables for HDL, TC, LDL and TG; and sex as classification variable and age as covariable for HT_O_, WT and BMI_O_. The SNP coding in **W**_α_ and **W**_δ_ is the same as the quantitative genetics coding for SNPs (Da et al., 2014), and the haplotype coding in **W**_αh_ is the same as the multi-allelic coding based on genetic partition (Da, 2015). The first moment is E(**y**) = **Xb**, and the second moments resulting from the reparameterized and equivalent model are:

(2) $$\text{Var}(a)=\sigma_{\text{α}}^{2}A_{g}=\sigma_{\text{α}}^{2}W_{\text{α}}W\prime_{\text{α}}/{\text{k}}_{\text{α}}=G_{\text{a}}$$

(3) $$\text{Var}(d)=\sigma_{\text{δ}}^{2}D_{g}=\sigma_{\text{δ}}^{2}W_{\text{δ}}W\prime_{\text{δ}}/{\text{k}}_{\text{δ}}=G_{\text{d}}$$

(4) $$\text{Var}(a_{\text{h}})=\sigma_{\text{αh}}^{2}A_{\text{gh}}=\sigma_{\text{αh}}^{2}W_{\text{αh}}W\prime_{\text{αh}}/{\text{k}}_{\text{αh}}=G_{\text{αh}}$$

(5) $$\begin{array}{l} \text{Var}(y)=Z(\sigma_{\text{α}}^{2}A_{\text{g}}+=\sigma_{\text{δ}}^{2}D_{\text{g}}+\sigma_{\text{αh}}^{2}A_{\text{αh}})Z\prime +{\text{σ}}_{\text{e}}^{2}I_{\text{N}}\\ =Z\text{(}G_{\text{a}}+G_{\text{d}}+G_{ah})Z\prime +{\text{σ}}_{\text{e}}^{2}I_{\text{N}}=V \end{array}$$

where $\sigma_{\alpha }^{2}$, $\sigma_{\delta }^{2}$ and $\sigma_{\alpha \,h}^{2}$ are the SNP additive variance, SNP dominance variance and haplotype additive variance, respectively, under the reparameterized and equivalent model; **A**_g_ = ${\text{W}}_{\alpha }\,{\text{W}}_{\alpha }^{\prime }$/k_α_ = SNP genomic additive relationship matrix; **D**_g_ = ${\text{W}}_{\delta }\,{\text{W}}_{\delta }^{\prime }/{\text{k}}_{\delta }$ = SNP genomic dominance relationship matrix; **A**_gh_ = ${\text{W}}_{\alpha \,h}\,{\text{W}}_{\alpha \,h}^{\prime }/{\text{k}}_{\alpha \,h}$ = haplotype genomic additive relationship matrix; k_α_ = $\text{tr}\left({\text{W}}_{\alpha }{\text{W}}_{\alpha }^{\prime }\right)/$n; k_δ_ = $\text{tr}\left({\text{W}}_{\delta }{\text{W}}_{\delta }^{\prime }\right)/$n; k_αh_ = $\text{tr}\left({\text{W}}_{\alpha \,h}{\text{W}}_{\alpha \,h}^{\prime }\right)/$n; $\sigma_{\text{e}}^{2}$ = residual variance; and **V** = phenotypic variance-covariance matrix. The GVCHAP program (Prakapenka et al., 2020) first calculates the variance components of $\sigma_{\alpha }^{2}$, $\sigma_{\delta }^{2}$ and $\sigma_{\alpha \,h}^{2}$ in Eqs. 2–5, and calculates GBLUP and associated reliability estimates as well as the heritability of each SNP and each haplotype block at the end of the GREML iterations.

### Validation Studies for Observed Prediction Accuracy of Haplotype Models

A 10-fold validation study was used to evaluate the prediction accuracy for each model. Individuals with phenotypic observations was randomly divided into 10 validation populations. The first nine validation populations had equal sample size and the tenth population generally had fewer observation than the first nine population. In each validation population, phenotypes were omitted when calculating GBLUP. For each method of haplotype blocking and each validation population, six prediction models were evaluated:

Model 1: SNP additive, dominance and haplotype additive values;
Model 2: SNP and haplotype additive values;
Model 3: SNP dominance values and haplotype additive values;
Model 4: haplotype additive values only;
Model 5: SNP additive and dominance values;
Model 6: SNP additive values only.

Models 1–4 contain haplotype additive values, and Models 5–6 are SNP models. The comparison between Models 1–4 and Models 5–6 for prediction accuracy provides an estimate whether haplotypes improve the prediction accuracy. Multiple measures of expected and observed prediction accuracy exist (Legarra et al., 2008; Da et al., 2014; Tan et al., 2017; Da, 2019; Prakapenka et al., 2020), and each type of genetic value in Models 1–6 has its own expected and observed prediction accuracies (Prakapenka et al., 2020). For simplicity, prediction accuracy of any of the six prediction models in this article refers to the observed prediction accuracy calculated as the correlation between the phenotypic values and the GBLUP of the total genetic values as summation of genetic values in the prediction model in the validation individuals averaged over the ten validation populations for each phenotype and each prediction model.

### Estimation of Haplotype Epistasis Heritability

Haplotype epistasis heritability can be estimated using two methods, variance-base method (VBM) and heritability-based method (HBM). However, the VBM method may have numerical problems (Supplementary Text 1). Under the HBM method, haplotype epistasis heritability is defined as the difference between the genotypic heritability of the haplotype model and the genotypic heritability of the SNP model, and is a measure to study the reason for the improvement in prediction accuracy of haplotype models. The HBM method had an upward bias compared to the VBM method that could have the problem of numerical instability (Supplementary Text 1). However, SNP effects and heritability estimates used genomic additive relationships that could have accounted for some epistasis effects and resulted in underestimates of haplotype epistasis heritability. Such underestimates and the bias in the estimates by the HBM method should cancel each other to some degree. The estimate of haplotype epistasis heritability can be represented by one formula, i.e.,

(6) $${\hat{\text{h}}}_{\text{E}}^{2}={\hat{\text{h}}}_{\text{g}}^{2}-{\hat{\text{h}}}_{\text{s}}^{2}$$

where ${\hat{\text{h}}}_{\text{E}}^{2}$ = haplotype epistasis heritability, ${\hat{\text{h}}}_{\text{g}}^{2}$ = total heritability from a prediction model with haplotypes (Models 1–4), and ${\hat{\text{h}}}_{\text{s}}^{2}$ = total SNP heritability from the corresponding SNP model (Model 5 or 6). A haplotype or SNP heritability is available from more than one model, and the contents of ${\hat{\text{h}}}_{\text{g}}^{2}$ and ${\hat{\text{h}}}_{\text{s}}^{2}$ from different models generally are different. Consequently, the correct use of Eq. 6 needs to consider the specific prediction model from which haplotype and SNP heritabilities were estimated. Let ${\hat{\text{h}}}_{\alpha \,1}^{2}$ = SNP additive heritability estimate from Model 6 with SNP additive values only, ${\hat{\text{h}}}_{\alpha \,2}^{2}$ = SNP additive heritability estimate from Model 5 with SNP additive and dominance values, **${\hat{\text{h}}}_{\delta }^{2}$** = SNP dominance heritability estimate from Model 5 with SNP additive and dominance values, ${\hat{\text{h}}}_{\alpha \,s}^{2}$ = SNP additive heritability estimate from a haplotype model with SNP and haplotype additive values (Model 1 or 2), **${\hat{\text{h}}}_{\delta \,s}^{2}$** = SNP dominance heritability estimate from a haplotype model with SNP dominance values (Model 1 or 3), and **${\hat{\text{h}}}_{\alpha \,h}^{2}$** = haplotype additive heritability estimate from any model with haplotypes (Models 1–4). Depending on the prediction model, ${\hat{\text{h}}}_{\text{g}}^{2}$ and ${\hat{\text{h}}}_{\text{s}}^{2}$ in Eq. 6 each has one of the following expressions:

(7) $${\hat{\text{h}}}_{\text{g}}^{2}={\hat{\text{h}}}_{\alpha \,h}^{2}\text{ for Model 4}$$

(8) $${\hat{\text{h}}}_{\text{g}}^{2}={\hat{\text{h}}}_{\alpha \,s}^{2}+{\hat{\text{h}}}_{\alpha \,h}^{2}\text{ for Model 2}$$

(9) $${\hat{\text{h}}}_{\text{g}}^{2}={\hat{\text{h}}}_{\alpha \,s}^{2}+{\hat{\text{h}}}_{\delta \,s}^{2}+{\hat{\text{h}}}_{\alpha \,h}^{2}\text{ for Model 1}$$

(10) $${\hat{\text{h}}}_{\text{g}}^{2}={\hat{\text{h}}}_{\delta \,s}^{2}+{\hat{\text{h}}}_{\alpha \,h}^{2}\text{ for Model 3}$$

(11) $${\hat{\text{h}}}_{\text{s}}^{2}={\hat{\text{h}}}_{\alpha \,1}^{2}\text{ for Model 6}$$

(12) $${\hat{\text{h}}}_{\text{s}}^{2}={\hat{\text{h}}}_{\alpha \,2}^{2}+{\hat{\text{h}}}_{\delta }^{2}\text{ for Model 5}$$

Then, estimate of haplotype epistasis heritability of Eq. 6 for a specific haplotype prediction model can be expressed as one of the following formulae:

(13) $${\hat{\text{h}}}_{\text{E}}^{2}={\hat{\text{h}}}_{\text{g}}^{2}-{\hat{\text{h}}}_{\text{s}}^{2}={\hat{\text{h}}}_{\alpha \,h}^{2}-{\hat{\text{h}}}_{\alpha \,1}^{2}\text{ for Model 4}$$

(14) $${\hat{\text{h}}}_{\text{E}}^{2}={\hat{\text{h}}}_{\text{g}}^{2}-{\hat{\text{h}}}_{\text{s}}^{2}=\left({\hat{\text{h}}}_{\alpha \,h}^{2}+{\hat{\text{h}}}_{\alpha \,s}^{2}\right)-{\hat{\text{h}}}_{\alpha \,1}^{2}\text{ for Model 2}$$

(15) $${\hat{\text{h}}}_{\text{E}}^{2}={\hat{\text{h}}}_{\text{g}}^{2}-{\hat{\text{h}}}_{\text{s}}^{2}=\left({\hat{\text{h}}}_{\alpha \,h}^{2}+{\hat{\text{h}}}_{\alpha \,s}^{2}+{\hat{\text{h}}}_{\delta \,s}^{2}\right)-\left({\hat{\text{h}}}_{\alpha \,2}^{2}+{\hat{\text{h}}}_{\delta }^{2}\right)\text{ for Model 1}$$

(16) $${\hat{\text{h}}}_{\text{E}}^{2}={\hat{\text{h}}}_{\text{g}}^{2}-{\hat{\text{h}}}_{\text{s}}^{2}=\left({\hat{\text{h}}}_{\alpha \,h}^{2}+{\hat{\text{h}}}_{\delta \,s}^{2}\right)-\left({\hat{\text{h}}}_{\alpha \,2}^{2}+{\hat{\text{h}}}_{\delta }^{2}\right)\text{ for Model 3}$$

The heritability estimates on the right-hand sides of Eqs. 7–16 are available from the GREML output files of GVCHAP (Prakapenka et al., 2020). Relative haplotype epistasis heritability is defined as the ratio of the haplotype epistasis heritability to the SNP additive heritability, as a measure of the size of haplotype epistasis heritability relative to SNP additive heritability. Depending on the prediction model with haplotypes, estimated relative haplotype epistasis heritability is:

(17) $${\hat{\text{h}}}_{\text{Er}}^{2}={\hat{\text{h}}}_{\text{E}}^{2}/{\hat{\text{h}}}_{\alpha \,1}^{2}\text{ for Models 2 and 4}$$

(18) $${\hat{\text{h}}}_{\text{Er}}^{2}={\hat{\text{h}}}_{\text{E}}^{2}/{\hat{\text{h}}}_{\alpha \,2}^{2}\text{ for Models 1 and 3}$$

The method of estimating haplotype heritability described above is based on the theoretical result of invariance property of GBLUP and GREML to repeated SNPs (Da et al., 2016; Tan et al., 2017) and the hypothesized haplotype model that a haplotype additive value is the summation of the SNP additive values and a haplotype epistasis value within the haplotype, plus a potential haplotype loss of additive values (Da et al., 2016), where haplotype epistasis effects between SNPs could include pairwise and high-order epistasis effects within the haplotype. The epistasis values in each haplotype block could only be additive × additive (A × A) epistasis values involving interaction between additive SNP values and could not involve dominance values because haplotype dominance values were not included in any of the prediction models. Based on this hypothesized haplotype model, haplotype additive values can be expressed as:

(19) $${\text{a}}_{\text{h}}=\text{a}+\varepsilon_{\text{h}}+\tau_{\text{h}}\approx \text{a}+\varepsilon_{\text{h}}$$

where **a**_h_ = n × 1 haplotype genomic additive values, **a** = n × 1 SNP genomic additive values, ***ε*_h_** = n × 1 haplotype epistasis values, and ***τ*_h_** = n × 1 haplotype loss of additive values based on empirical evidence that haplotype-only model was less accurate than SNP model in some cases. According to the invariance property that GBLUP and GREML are unaffected by duplicate SNPs (Da et al., 2016; Tan et al., 2017), the combination of SNP additive values (**a**_s_) and haplotype additive values approximately predicts the summation of SNP additive values and haplotype epistasis effects as defined by Eq. 19, i.e.,

(20) $${\text{a}}_{\text{s}}+{\text{a}}_{\text{h}}\approx \text{a}+\varepsilon_{\text{h}}$$

Eqs. 19 and 20 are the foundation for estimating haplotype epistasis heritability using Eqs. 13–16. With haplotype loss, estimates of haplotype epistasis heritability using Eqs. 13–16 underestimate the true epistasis heritability. Haplotype loss likely is due to the less accurate estimates of SNP effects by the haplotype model than by a SNP model, and likely is the reason why the combination of SNP and haplotype values in the prediction model can increase the prediction accuracy, as shown in this study. Without haplotype loss, haplotype additive values accurately estimate SNP additive values and the inclusion of SNP additive values in the prediction model is not helpful, as shown in this study.

### Profiles of Heritability Estimates of SNPs and Haplotype Blocks

A heritability profile in this research is a Manhattan plot of heritability estimates of SNPs or haplotype blocks using the SNPEVG2 program (Wang et al., 2012). The heritability size of each SNP is related to the number of SNPs in the model, the larger the number of SNPs, the smaller each SNP heritability (Da et al., 2016; Tan et al., 2017). Therefore, the heritability of a SNP is not comparable with the heritability of a haplotype block. However, each heritability profile of SNPs or haplotypes provides a global view of relative genetic contributions of different genes and chromosome locations to the phenotype, and such global views of relative genetics contributions are comparable between SNPs and haplotypes. The differences between heritability profiles of SNP and haplotypes provide indications about the likely reason why a haplotype model did or did not improve the prediction accuracy.

## Results and Discussion

**FIGURE 1:**
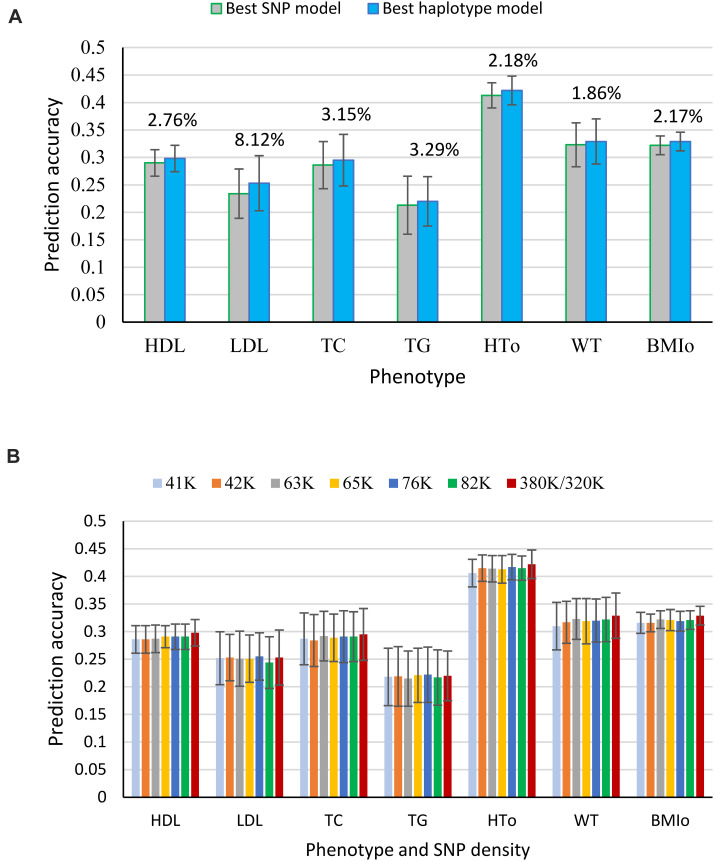
Prediction accuracy of haplotype prediction models. **(A)** Average prediction accuracy of the best haplotype model relative to the best SNP model for each phenotype from ten-fold validations. **(B)** Average prediction accuracy of the best haplotype model from ten-fold validations for each SNP density. The error bar is one standard deviation above and below the average prediction accuracy, where standard deviation was calculated from ten-fold validations.

**FIGURE 2:**
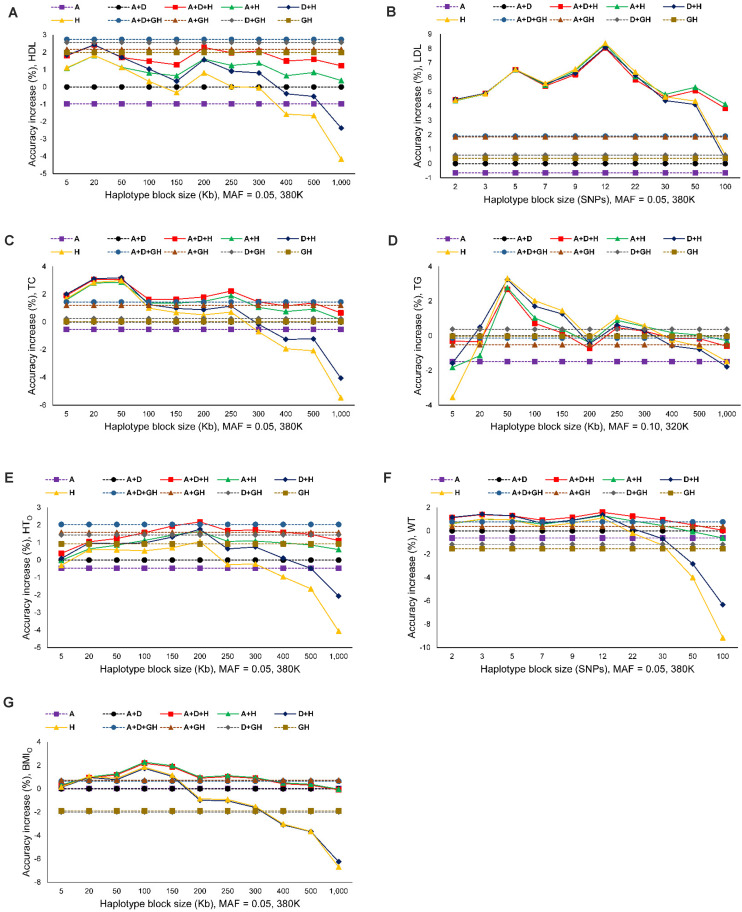
Prediction accuracy of best haplotype models relative to the SNP models and gene-based haplotype models. **(A)** High-density lipoproteins (HDL). **(B)** Low-density lipoproteins (LDL). **(C)** Total cholesterol (TC). **(D)** Triglycerides (TG). **(E)** Height using the original phenotypic observations (HT_O_). **(F)** Weight (WT). **(G)** Body mass index using the original phenotypic observations (BMI_O_). A = SNP additive values. D = SNP dominance values. H = haplotype additive values. GH = haplotype additive values of genes.

The seven phenotypes including the original and normality transformed triglycerides all had improved prediction accuracy due to haplotypes in the prediction model. The increase in prediction accuracy due to haplotypes for the best haplotype models relative to the prediction accuracy of the best SNP model (additive only, or additive and dominance) was in the range of 1.86-8.12% (Figure 1A and Table 4). Figure 1A is a summary of prediction accuracy for the best haplotype models, whereas Table 4 has detailed information about accuracy increases of the best haplotype models. The eight different SNP densities with 40,941-380,705 SNPs (Supplementary Table 1) had similar prediction accuracies (Figure 1B). Figure 2 shows the entire range of haplotype blocks with the best prediction models, including 5-1000 Kb blocks, and 2-SNP to 100-SNP blocks with comparison to two SNP models and four gene-based haplotype models. The haplotype blocking method using gene boundaries had the best prediction model for one phenotype (HDL), fixed chromosome distances had the best prediction model for four phenotypes, and fixed number of SNPs had the best prediction model for three phenotypes including the original triglycerides (Table 4). The summary below focuses on the results of the best prediction models using the 380K SNP set with MAF of 0.05 and 320K SNP set with MAF of 0.10, and the complete results for each haplotype blocking method are shown in Supplementary Figures 4–7.

**TABLE 4:** Accuracy increase of the best prediction models with haplotype additive values relative to the best single-SNP model.

Trait	HDL	LDL	TC	TG_O_	TG	HT_O_	WT	BMI_O_
**SNP prediction accuracy (MAF = 0.05)**								
Additive only (A), mean ± SD	0.287 ± 0.024	0.232 ± 0.045	0.285 ± 0.041	**0.152** ± 0.074	0.210 ± 0.048	0.411 ± 0.024	0.322 ± 0.040	0.322 ± 0.017
Additive and dominance (A + D), mean ± SD	**0.290** ± 0.022	**0.234** ± 0.045	**0.286** ± 0.043	0.150 ± 0.076	**0.213** ± 0.053	**0.413** ± 0.023	**0.323** ± 0.040	**0.322** ± 0.017
% Accuracy increase of (A + D) over (A)	1.05	0.86	0.35	-1.32	1.43	0.49	0.31	0.00
**SNP prediction accuracy (MAF = 0.10)**								
Additive only (A), mean ± SD	0.284 ± 0.024	0.232 ± 0.045	0.283 ± 0.040	0.152 ± 0.075	0.209 ± 0.048	0.408 ± 0.024	0.318 ± 0.040	0.320 ± 0.017
Additive and dominance (A + D), mean ± SD	0.287 ± 0.022	0.233 ± 0.045	0.285 ± 0.042	0.151 ± 0.077	0.213 ± 0.053	0.411 ± 0.023	0.321 ± 0.039	0.320 ± 0.017
**Haplotype prediction accuracy**								
MAF	0.05	0.05	0.05	0.10	0.10	0.05	0.05	0.05
Best prediction model	A + D + H	H	D + H	H	H	A + D + H	A + D + H	A + H
Best haplotype blocking method	Genes	12 SNPs	50 Kb	3 SNPs	50 Kb	200 Kb	12 SNPs	100 Kb
Prediction accuracy (mean ± SD)	0.298 ± 0.024	0.253 ± 0.050	0.295 ± 0.047	0.156 ± 0.073	0.220 ± 0.045	0.422 ± 0.026	0.329 ± 0.041	0.329 ± 0.017
% Accuracy increase over best SNP model	2.76	8.12	3.15	3.31	3.29	2.18	1.86	2.17

*Accuracy increase is the percentage increase in observed prediction accuracy of the best haplotype model relative to the accuracy of the best SNP model (in bold face). SD, standard deviation; A, SNP additive values; D, SNP dominance values; H, haplotype additive values; MAF, minor allele frequency.*

### Prediction Accuracy of Haplotype Models for Cholesterol Phenotypes

For the three cholesterol phenotypes, HDL had the highest prediction accuracy (0.298), followed by TC (0.295) and LDL (0.253). However, LDL had the largest accuracy increase due to haplotypes, 8.12% accuracy increase over the best SNP model, followed by TC (3.15%) and HDL (2.76%). The best prediction model was Model 1 with SNP additive and dominance values and haplotype additive values using autosome gene boundaries as haplotype blocks for HDL, Model 4 with haplotype additive values using 12-SNP haplotype blocks for LDL, and Model 3 with SNP dominance values and haplotype additive values using 50-Kb haplotype blocks for TC (Figures 1A, 2A–C and Table 4).

### Prediction Accuracy of Haplotype Models for Triglycerides (TG)

Among the seven phenotypes, TG_O_ had the most skewed distribution (Supplementary Figure 2) and benefited most for prediction accuracy from the normality transformation. The prediction accuracy of the best SNP model (Model 5) for the normality transformed TG was 0.213 compared to 0.152 for the best SNP model (Model 6) for TG_O_, a 40.13% increase in prediction accuracy of TG over that of TG_O_. Haplotype additive model using 50-Kb blocks further increased the prediction accuracy of TG by 3.29% over the haplotype additive model using 3-SNP blocks for TG_O_. Compared to the SNP additive model for TG_O_, the combination of normality transformation and haplotype additive values achieved 44.47% increase in prediction accuracy over the best SNP prediction model for TG_O_ (Figures 1A, 2D and Table 4).

### Prediction Accuracy of Haplotype Models for Body Phenotypes

The prediction accuracy was 0.422 for HT_O_, 0.329 for WT and BMI_O_ (Figures 1A, 2E–G and Table 4). HT_O_ had the highest prediction accuracy (0.422) and the highest total heritability (≈ 100%, Table 5) among the seven phenotypes. Increase in prediction accuracy due to haplotypes relative to the best SNP prediction model was 2.18% for HT_O_, 1.86% for WT, and 2.17% for BMI_O_. The best prediction model was the full model with SNP additive and dominance values and haplotype additive values (Model 1) for HT_O_ and WT, and Model 2 with SNP and haplotype additive values for BMI_O_, but the best method for haplotype blocking was 200-Kb blocks for HT_O_, 12-SNP blocks for WT and 100-Kb blocks for BMI_O_ (Table 5). Compared to the three cholesterol phenotypes (HDL, LDL, TC) and TG, the three body phenotypes had higher prediction accuracy but lower percentage accuracy increase due to haplotypes because of the higher prediction accuracy of the SNP models.

**TABLE 5:** Relationship between haplotype epistasis heritability and prediction accuracy for best haplotype prediction models.

Trait	HDL	LDL	TC	TG	HT_O_	Weight	BMI_O_
**SNP model with additive values (A), MAF = 0.05**							
Additive heritability (${\hat{\text{h}}}_{\mathrm{s1}}^{2}$ = ${\hat{\text{h}}}_{\alpha \,1}^{2}$)	0.409	0.469	0.409	0.312	0.773	0.493	0.424
**SNP model with additive values (A), MAF = 0.10**							
Additive heritability (${\hat{\text{h}}}_{\mathrm{s1}}^{2}$ = ${\hat{\text{h}}}_{\alpha \,1}^{2}$)	0.401	0.464	0.402	0.308	0.760	0.483	0.417
**SNP model with additive and dominance values (A + D), MAF = 0.05**							
Additive heritability (${\hat{\text{h}}}_{\alpha \,2}^{2}$)	0.386	0.406	0.389	0.260	0.739	0.474	0.415
Dominance heritability (${\hat{\text{h}}}_{\delta }^{2}$)	0.121	0.174	0.102	0.125	0.198	0.088	0.044
SNP total heritability (${\hat{\text{h}}}_{\text{s}}^{2}$)	0.507	0.580	0.491	0.385	0.937	0.562	0.459
**SNP model with additive and dominance values (A + D), MAF = 0.10**							
Additive heritability (${\hat{\text{h}}}_{\alpha \,2}^{2}$)	0.378	0.404	0.381	0.257	0.724	0.462	0.407
Dominance heritability (${\hat{\text{h}}}_{\delta }^{2}$)	0.122	0.170	0.107	0.126	0.212	0.098	0.049
SNP total heritability (${\hat{\text{h}}}_{\text{s}}^{2}$)	0.500	0.574	0.488	0.382	0.935	0.560	0.456
**Haplotype prediction models**							
MAF	0.05	0.05	0.05	0.10	0.05	0.05	0.05
Best prediction model	A + D + H	H	D + H	H	A + D + H	A + D + H	A + H
Best haplotype blocking method	Genes	12 SNPs	50 Kb	50 Kb	200 Kb	12 SNPs	100 Kb
Accuracy increase (%)	2.76	8.12	3.15	3.29	2.18	1.86	2.17
SNP additive heritability (${\hat{\text{h}}}_{\alpha \,s}^{2}$)	0.070	-	-	-	0.359	0.124	0.123
SNP dominance heritability (${\hat{\text{h}}}_{\delta \,s}^{2}$)	0.094	-	0.077	-	0.147	0.057	-
Haplotype additive heritability (${\hat{\text{h}}}_{\alpha \,h}^{2}$)	0.394	0.616	0.452	0.353	0.494	0.422	0.366
Total heritability (${\hat{\text{h}}}_{\text{g}}^{2}$)	0.558	0.616	0.530	0.353	0.999	0.603	0.488
**Estimates of haplotype epistasis heritability**							
Haplotype epistasis heritability (${\hat{\text{h}}}_{\text{E}}^{2}$)	0.051	0.147	0.039	0.045	0.062	0.041	0.064
Relative haplotype epistasis heritability (${\hat{\text{h}}}_{\text{Er}}^{2}$,%)	13.27	31.27	10.03	14.53	8.90	8.65	15.05

*Accuracy increase is the percentage increase in observed prediction accuracy of the best haplotype model relative to the accuracy of the best SNP model (in bold face). A, SNP additive values; D, SNP dominance values; H, haplotype additive values; MAF, minor allele frequency; SNP total heritability is the sum of SNP additive and dominance heritabilities (Eq. 12). Total heritability is the sum of all heritabilities of genetic values in the model with haplotypes (Eqs. 7–10). Haplotype epistasis heritability was calculated using Eqs. 13–16. Relative haplotype epistasis heritability was calculated using Eqs. 17 and 18.*

### SNP and Haplotype Heritability Estimates

**FIGURE 3:**
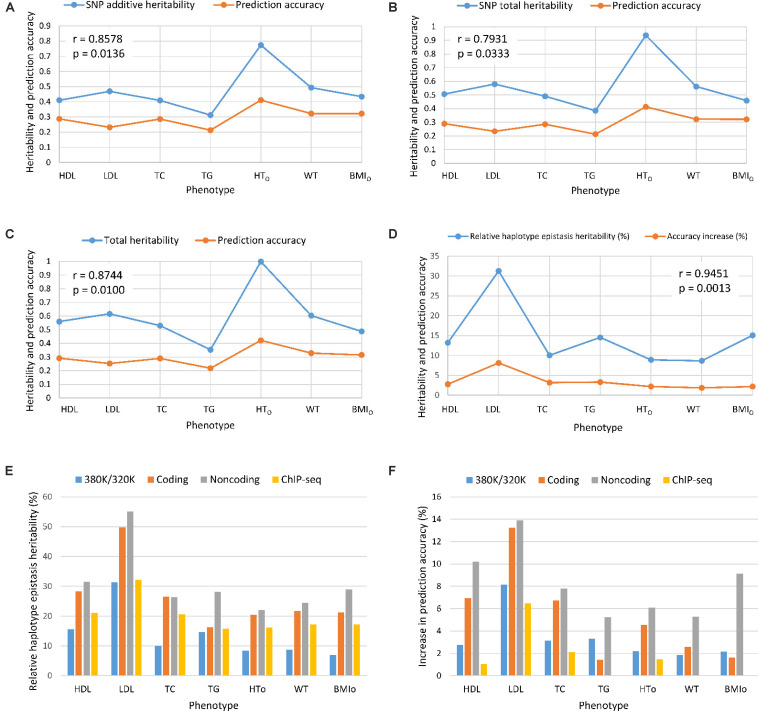
Prediction accuracy had strong concordance with the size of heritability estimates. **(A)** SNP additive heritability and prediction accuracy. **(B)** SNP total heritability and prediction accuracy. **(C)** Prediction accuracy and total heritability as summation of all heritabilities of the prediction model. **(D)** Relative haplotype epistasis heritability and accuracy increase. **(E)** Relative haplotype epistasis heritability estimates in functional regions. **(F)** Accuracy increase due to haplotypes relative to SNPs in functional regions. r = correlation coefficient. p = probability that the null hypothesis of correlation is true = significance level.

From the 380K SNP set with MAF of 0.05, additive heritability estimate from the SNP additive model (Model 6) was in the range of 0.312 for TG to 0.773 for HT_O_, and was 0.260 for TG and 0.739 for HT_O_ from the SNP model with additive and dominance values (Model 5), showing that the inclusion of dominance values in the prediction model decreased the additive heritability for all phenotypes (Table 5). Dominance heritability was in the range of 0.044 for BMI_O_ to 0.198 for HT_O_. However, dominance values resulted in no increase in prediction accuracy for TG, TC and BMI_O_, and less than 1% increases for LDL, HT_O_ and WT. HDL had the largest accuracy increase of 1.05% (0.290 versus 0.287) due to dominance values over the SNP additive model (Table 4). Haplotype models had higher total heritability as the sum of heritability estimates of all genetic values in the prediction model (Eqs. 7–10) than those of SNP models (Eqs. 11 and 12) for all phenotypes. The sizes of SNP additive heritability, haplotype heritability and total heritability were in concordance with the prediction accuracy except LDL (Figures 3A–C). The lack of concordance between heritability and accuracy for LDL was unknown but could be related to the small sample size of LDL, noting that TG also had similarly small sample size that was about half of the sample sizes of the other five phenotypes. Across the seven phenotypes, SNP additive heritability, haplotype heritability and total heritability were significantly correlated with the prediction accuracy (p = 0.010 to 0.033, Figures 3A–C). The fact that haplotype models had higher total heritability than SNP models was due to the presence of haplotype epistasis values hypothesized by Eqs. 19 and 20 and shown by the analysis below.

### Haplotype Epistasis Heritability and Haplotype Prediction Accuracy

Estimates of haplotype epistasis heritability using Eqs. 13–16 and estimates of relative haplotype epistasis heritability using Eqs. 17 and 18 indicated that epistasis effects within haplotype blocks was the reason for the increased prediction accuracy from haplotypes. Haplotype epistasis heritability was in the range of 0.039-0.147, and relative haplotype epistasis heritability was in the range of 8.65-31.27% of the SNP additive heritability. LDL had the largest haplotype epistasis heritability of 0.147 and the largest relative epistasis heritability of 31.27% (Table 5), i.e., the haplotype epistasis heritability explained 14.7% of the phenotypic variance and was 31.27% of the SNP additive heritability. This largest haplotype epistasis heritability was accompanied by the largest increase in prediction accuracy due to haplotypes (8.12%) relative to the prediction accuracy of the best SNP model, Model 6 with SNP additive values only. The other two cholesterol phenotypes (HDL and TC) and TG all had higher haplotype epistasis heritability estimates and larger increases in prediction accuracy due to haplotypes than the three body phenotypes (HT_O_, WT, and BMI_O_). In general, haplotype epistasis heritability estimates were in concordance with accuracy increases due to haplotypes except BMI_O_, and were strongly correlated with the prediction accuracy across the seven phenotypes (p = 0.0013, Figure 3D), noting that this correlation was more significant than those between prediction accuracy and SNP additive heritability, haplotype heritability and total heritability (Figures 3A–C). Since haplotype epistasis is the only difference between haplotype and SNP additive values in the absence of haplotype loss (Eq. 20), haplotype epistasis is the reason for the increased prediction accuracy of haplotype models. Haplotype loss defined in Eq. 19 also affects the prediction accuracy of haplotype models, but the inclusion of SNP additive values compensates the haplotype loss as shown later. Consequently, haplotype epistasis is the only reason for the increased prediction accuracy due to haplotypes with or without haplotype loss.

### Autosome Genes, Coding, and Non-coding Genes, ChIP-seq

Given the presence of haplotype epistasis calculated from all autosomes for all seven traits, we investigated whether functional regions had stronger haplotype epistasis, including coding and noncoding genes, and ChIP-seq sites. SNPs and haplotypes for these functional regions were extracted from the 380K SNPs and haplotypes with MAF of 0.05. The results showed that the functional regions had much stronger haplotype epistasis than observed for all autosomes. The relative haplotype epistasis heritability calculated from all autosomes was 8.65-31.27% (above 15% only for HDL and LDL) (Figure 3D), and 15-55.07% calculated from haplotype and SNP heritabilities in the functional regions (Figure 3E). Other than TC, noncoding genes (mostly lncRNAs) had the highest relative haplotype epistasis heritability, followed by coding genes, ChIP-seq, and all autosomes. These results showed that functional regions had strong haplotype epistasis affecting the phenotypes, relative to the SNP effects in those regions. The analysis of accuracy increases from haplotype additive values relative to SNP values showed haplotypes mostly had higher prediction accuracy than SNPs in the functional regions (Figure 3F). Haplotypes of noncoding genes had the largest increases in prediction accuracy relative to SNPs in the noncoding genes for all seven phenotypes, followed by coding genes for five phenotypes, the best prediction models using all autosomes (380K/320K), and ChIP-seq. The results of noncoding genes indicated that haplotype epistasis effects likely were the typical effects of noncoding genes. Although such relative accuracy increases provided an understanding about the role of functional regions in prediction accuracy, such measures had limitations, e.g., ChIP-seq had high prediction accuracy but low relative accuracy increase for LDL. To overcome such limitations, we further compared the prediction accuracies of the best prediction models in functional regions with the best prediction models using the 380K/320K SNP sets (Table 5).

**FIGURE 4:**
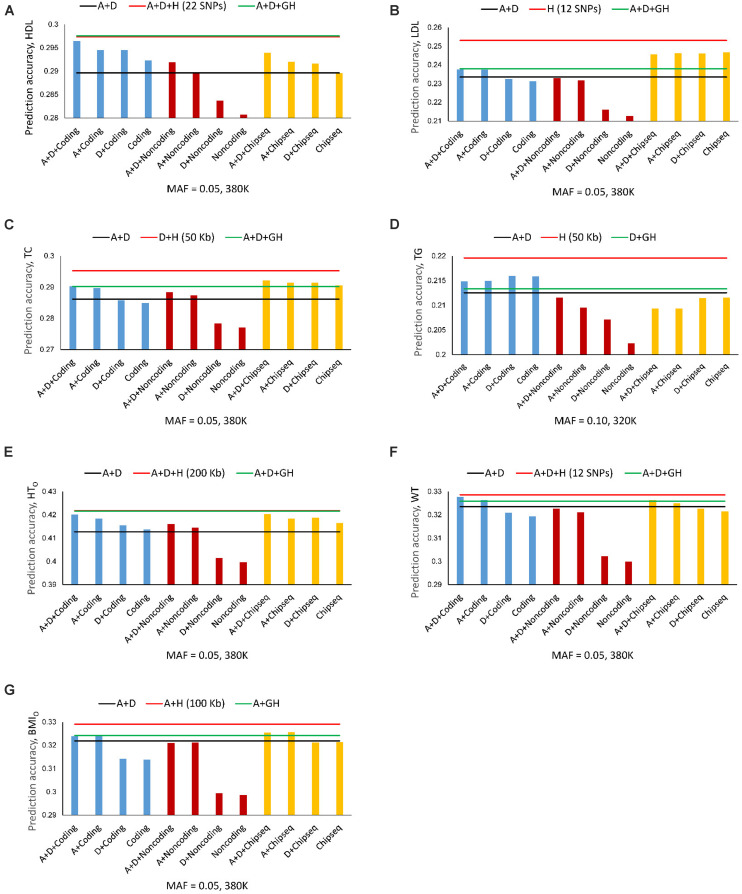
Average prediction accuracy of coding genes, noncoding genes and ChIP-seq from ten-fold validations relative to the prediction accuracy of SNP models with additive and dominance values (black line), the best autosome gene haplotype model (green line), and the overall best haplotype model (red line). **(A)** High-density lipoproteins (HDL). **(B)** Low-density lipoproteins (LDL). **(C)** Total cholesterol (TC). **(D)** Triglycerides (TG). **(E)** Height using the original phenotypic observations (HT_O_). The red and green lines overlap. The red line is slightly higher but is invisible. **(F)** Weight (WT). **(G)** Body mass index using the original phenotypic observations (BMI_O_). A = SNP additive values. D = SNP dominance values. H = haplotype additive values. GH = haplotype additive values of genes.

The best prediction models using autosome genes and the 380K/320K still had the highest prediction accuracy for all seven phenotypes (Figures 4A–G). The full model (Model 1) with gene boundaries as haplotype blocks was the best prediction model for HDL (Figure 2A and Table 4), but was only slightly better than the full model with 22-SNP blocks (Figure 4A), and was virtually tied with the full model using 200 Kb blocks for HT_O_ (Figure 4E). Coding genes performed better than all autosome genes for TG (Figure 4D) and WT (Figure 4F), and almost had the same accuracy as the best prediction models for HDL, HT_O_, and BMI_O_ (Figures 4A,E,F). The accuracy of the noncoding genes (mostly lncRNAs) was not as high as that of the coding genes, but the combination of haplotype additive values in noncoding genes with SNP additive and dominance values had better accuracy than the best SNP models for HDL, TC, and HT_O_ (Figures 4A,C,E), indicating that noncoding genes were relevant to the accuracy of genomic prediction. ChIP-seq had better prediction accuracy than all autosome genes for LDL, TC, and BMI_O_ (Figures 4B,C,G), and had better prediction accuracy than the best SNP models when combined with SNP additive and dominance values for six of the seven phenotypes, with TG being the only exception. The ChIP-seq accuracy was most striking for LDL, where all four haplotype models using ChIP-seq sites as the haplotype blocks had similarly higher prediction accuracy than all autosome genes (Figure 4B), indicating that regulatory genetic mechanism likely was important for LDL. Among the three types of functional genomic information for defining haplotype blocks, coding genes were most important for the prediction accuracy of HDL, HT_O_ and WT, whereas ChIP-seq was most important for LDL, TC and BMI_O_. These results showed that functional genomic information is relevant to genomic prediction and that multi-allelic haplotype analysis can be a method to utilize functional genomic information or integrate functional and structural genomic information for genomic prediction.

### Comparison of SNP and Haplotype Heritability Profiles

**FIGURE 5:**
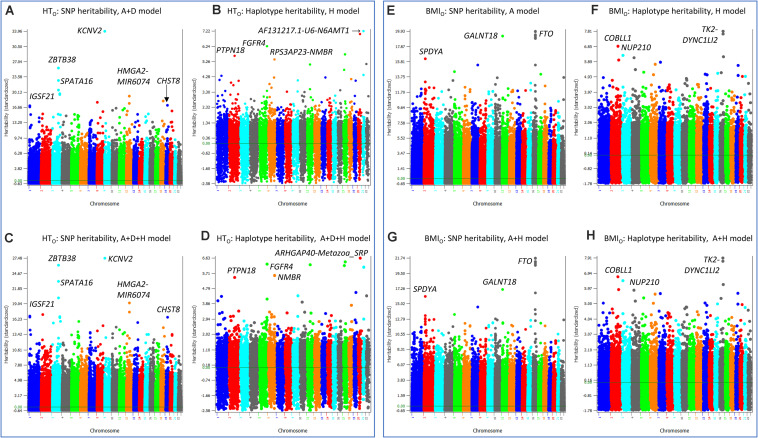
SNP and haplotype heritability profiles of height using the original phenotypic observations (HT_O_) and body mass index using the original phenotypic observations (BMI_O_). **(A)** SNP additive heritability estimates of HT_O_ from the A + D model (Model 5). **(B)** SNP additive heritability estimates of HT_O_ from the A + D + H model (Model 1). **(C)** Haplotype additive heritability estimates of HT_O_ from the haplotype-only model (Model 4). **(D)** Haplotype heritability estimates of HT_O_ from the A + D + H model (Model 1). **(E)** SNP additive heritability estimates of BMI_O_ from the SNP additive model (Model 6). **(F)** Haplotype additive heritability estimates of BMI_O_ from the haplotype-only model (Model 4). **(G)** SNP additive heritability estimates of BMI_O_ from the A + H model (Model 2). **(H)** Haplotype heritability estimates of BMI_O_ from the A + H model (Model 2). A = SNP additive values. D = SNP dominance values. H = haplotype additive values. GH = haplotype additive values of genes.

**FIGURE 6:**
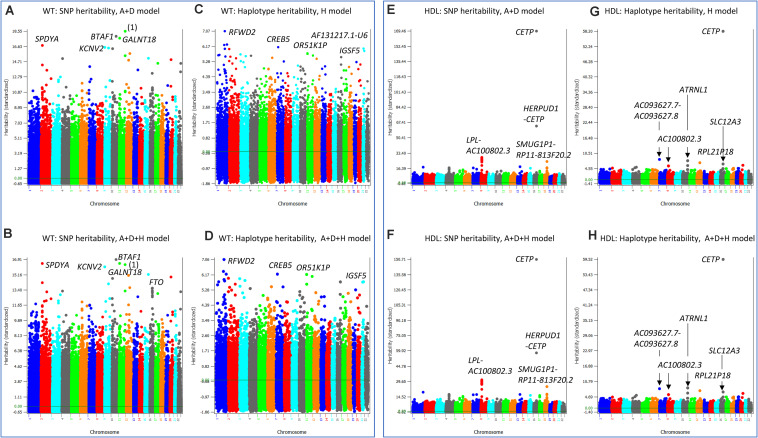
SNP and haplotype heritability profiles of weight (WT) and high-density lipoproteins (HDL). **(A)** SNP additive heritability estimates of WT from the A + D model (Model 5). ‘(1)’ represents RP11-469N6.1-RP11-555G19.1. **(B)** SNP additive heritability estimates of WT from the A + D + H model (Model 1). **(C)** Haplotype additive heritability estimates of WT from the haplotype-only model (Model 4). **(D)** Haplotype heritability estimates of WT from the A + D + H model (Model 1). **(E)** SNP additive heritability estimates of HDL from the A + D additive model (Model 5). **(F)** Haplotype additive heritability estimates of HDL from the haplotype-only model (Model 4). **(G)** SNP additive heritability estimates of HDL from the A + D + H model (Model 1). **(H)** Haplotype heritability estimates of HDL from the A + D + H model (Model 1). A = SNP additive values. D = SNP dominance values. H = haplotype additive values. GH = haplotype additive values of genes.

The similarities and differences between the SNP and haplotype heritability profiles may provide an understanding of the reasons for the success or failure of a haplotype prediction model. The most striking examples of not sharing the same genes or regions with the highest SNP and haplotype heritabilities were HT_O_ and BMI_O_. For HT_O_, SNP additive heritability profile from the SNP A + D model (Model 5) (Figure 5A) was similar to the SNP additive heritability profile from the A + D + H (Model 1) (Figure 5B), and haplotype heritability profile from the haplotype-only model (Figure 5C) was similar to the haplotype heritability profile from the A + D + H (Model 1) (Figure 5D). However, the two SNP heritability profiles (Figures 5A,B) all identified *KCNV2* and *ZBTB38* to have the highest heritability estimates, but the two haplotype heritability profiles (Figures 5C,D) did not identify these two genes to have high haplotype heritability. For BMI_O_, SNP additive heritability profile from the SNP model with additive values (Figure 5E) was similar to the SNP additive heritability profile from the model with SNP and haplotype additive values (Figure 5F), and haplotype heritability profile from the haplotype-only model (Figure 5G) was similar to the haplotype heritability profile from the full model (Model 1) (Figure 5H). The two SNP heritability profiles (Figures 5E,F) all identified *FTO*, *GALNT18* and *SPDYA* to have the highest heritability, but the two haplotype profiles (Figures 5G,H) did not identify these genes to have high haplotype heritability. For WT, genes with the highest SNP heritability (Figures 6A,B) were different from genes with the highest heritability profiles (Figures 6C,D). The inclusion of SNP additive values in the prediction model likely compensated the inaccuracy of the haplotype heritability estimates for the genes with the highest SNP heritability estimates, including *KCNV2* and *ZBTB38* for HT_O_, *FTO*, *GALNT18* and *SPDYA* for BMI_O_, and at least five genes for WT.

High density lipoproteins had the simplest patterns of the SNP and haplotype heritability profiles among the four phenotypes (HT_O_, BMI_O_, WT, HDL) with the A + D + H model (Model 1) as the best prediction model. The SNP heritability profiles from the A + D model (Model 5) (Figure 6E) and that from the A + D + H model (Model 1) (Figure 6F) were virtually identical, and the haplotype heritability profiles from the H model (Model 4) (Figure 6G) and that from the A + D + H model (Model 1) (Figure 6H) were also virtually identical. The *CETP* gene had the highest SNP and haplotype additive heritability estimates (Figures 6E–H). The *LPL-AC100802.3* region had the second highest SNP heritability estimates (Figure 6E) but did not have the second highest haplotype heritability estimates in the same region (Figure 6H). This difference likely contributed to the higher prediction accuracy with SNP additive values in the prediction model than without. Several regions including the *ATRNL1* gene had high haplotype heritability but not high SNP heritability estimates, and such differences likely were due to the presence of haplotype epistasis effects in those regions.

**FIGURE 7:**
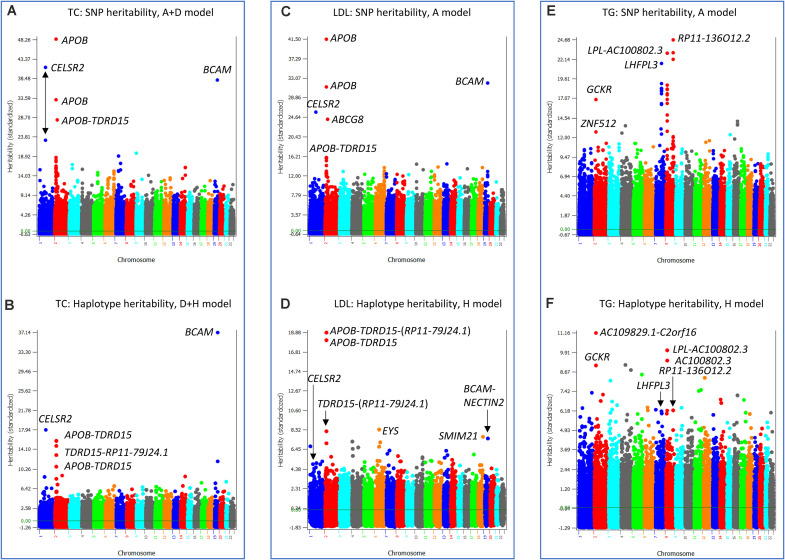
SNP and haplotype heritability profiles of total cholesterol (TC), low-density lipoproteins (LDL) and triglycerides (TG). **(A)** SNP additive heritability estimates of TC from the A + D model (Model 5). **(B)** Haplotype additive heritability estimates of TC from the model with haplotype additive values and SNP dominance values (Model 3). **(C)** SNP additive heritability estimates of LDL from the SNP additive model (Model 6). **(D)** Haplotype heritability estimates of LDL from the haplotype-only model (Model 4). **(E)** SNP additive heritability estimates of TG from the SNP additive model (Model 6). **(F)** Haplotype heritability estimates of TG from the haplotype-only model (Model 4). A = SNP additive values. D = SNP dominance values. H = haplotype additive values. GH = haplotype additive values of genes.

Three phenotypes had the best prediction model with haplotype additive values without SNP additive values, TC, LDL and TG. The best prediction model for TC was the D + H model (Model 3). The SNP heritability profile of TC (Figure 7A) showed that *APOB*, *CELSR2* and *BCAM* genes had the highest SNP heritability estimates, and these genes also had the highest haplotype heritability estimates (Figure 7B). Given that the inclusion of SNP additive values did not increase the prediction accuracy over the D + H model without SNP additive values, the haplotype additive values fully accounted for the SNP additive values. LDL and TG had the haplotype-only model (Model 4) as the best prediction model. Similar to the analysis for TC, the SNP heritabilities of LDL (Figure 7C) were fully accounted for by the haplotype heritabilities of LDL (Figure 7D), and the SNP heritabilities of TG (Figure 7E) were fully accounted for by the haplotype heritabilites of TG (Figure 7F).

The comparison of SNP and haplotype heritability profiles showed that haplotype loss was in the form of less accurate estimates of SNP heritabilities than those from the corresponding SNP models, and the inclusion of SNP additive values in the prediction model compensated the haplotype loss. The exact reason for haplotype loss was unknown, but incorrect size of haplotype blocks for chromosome regions with high SNP heritabilities such as excessively large blocks (>200 Kb blocks, Figures 2A,C–E,G) or excessively small blocks (5-Kb blocks, Figure 2D) could be a reason for incorrect estimates of SNP heritabilities by haplotype. Haplotype imputing errors in the regions with high SNP heritabilities could also fail to identify those regions with high haplotype heritabilities. For phenotypes with haplotype-only models as the best prediction models, haplotype loss was negligible or nonexistent, and haplotype effects more accurately estimated the SNP effects, given that adding SNP values decreased the prediction accuracy relative to the haplotype-only models.

### Effect of MAF on Haplotype Analysis

Haplotype analysis could be complicated by rare alleles that could generate many rare haplotypes, particularly when the number of SNPs is large in the haplotype block. In this case, increasing MAF could reduce the number of rare haplotypes. To evaluate the effect of increased MAF, we produced two high density SNP sets, MAF = 0.05 for the 380K and MAF = 0.10 for the 320K (Supplementary Table 1). The 320K indeed reduced the number of haplotypes relative to the 380K, e.g., the 320K had 61.46 SNPs (Supplementary Table 5) and the 380K had 71.30 SNPs on average for the 500 Kb haplotype blocks (Table 1). However, only TG (and TG_O_) had the best prediction model using the 320K, and the other six phenotypes had the best prediction models using the 380K (Table 4), indicating that reducing the number of haplotypes by increasing MAF did not achieve improved prediction accuracy for most phenotypes.

### Effect of SNP Density on Haplotype Analysis

To obtain indications about the minimal SNP density required and the suitability of medium SNP densities for haplotype genomic prediction, we evaluated eight SNP densities ranging from 40,941 to 380,705 SNPs (Supplementary Table 1) for prediction accuracy and heritability estimates (Supplementary Tables 8–15).

For prediction accuracy, we identified the best haplotype prediction model for each SNP set using a 10-fold validation study per model per phenotype (Supplementary Table 8). Although the different SNP densities had similar prediction accuracies (Figure 1B), the best haplotype block size increased as the SNP density decreased. For the example of HDL, the best block size was 250 Kb for the 82K set, 1000 Kb for the 63K set, and 1500 Kb for the 41K and 42K sets, and the other six phenotypes had similar trend though not as typical as HDL (Supplementary Table 8).

**FIGURE 8:**
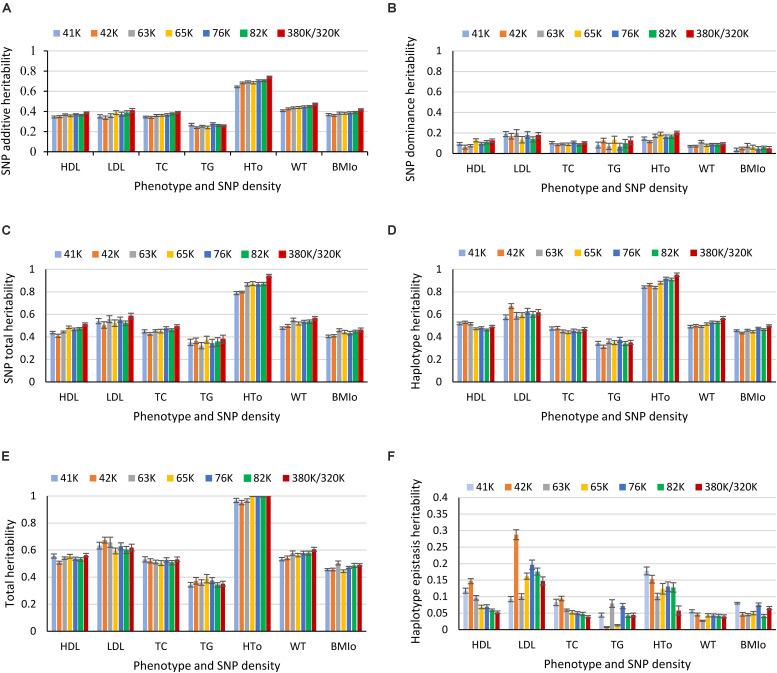
Average heritability estimate from ten-fold validations for each SNP density. **(A)** SNP additive heritability. **(B)** SNP dominance heritability. **(C)** SNP total heritability. **(D)** Haplotype heritability. **(E)** Total heritability as summation of from the haplotype heritability and SNP total heritability. **(F)** Haplotype epistasis heritability. The error bar is one standard deviation above and below the average heritability estimate, where standard deviation was calculated from the ten-fold validations.

Heritability estimates from the best prediction models of the eight SNP densities were mostly similar except a few visible differences. For HT_O_, the 380K SNP set had the highest estimates of SNP additive heritability (Figure 8A), SNP dominance heritability (Figure 8B), SNP total heritability (Figure 8C), haplotype additive heritability (Figure 8D), and total heritability of haplotypes and SNPs (Figure 8E). The 41K, 42K and 63K had large variations in estimates of haplotype epistasis heritability for LDL and the 42K and 65K had large variations for TG (Figure 8F). The estimates of haplotype epistasis heritability by the 76K, 82K, and 380K/320K were similar with the exception of HT_O_, for which the 380K had the lowest estimate of haplotype epistasis heritability due to the highest estimate of the SNP additive heritability (Figure 8F). Overall, results of various SNP densities were surprisingly similar for prediction accuracy (Figure 1B) and for heritability estimates except those of haplotype epistasis heritability (Figure 8). Based on the estimates of haplotype epistasis heritability, a SNP density of 76K or higher should be preferred for haplotype genomic prediction given the large variations in estimates of haplotype epistasis heritability by the 41K, 42K, 63K, and 65K.

**FIGURE 9:**
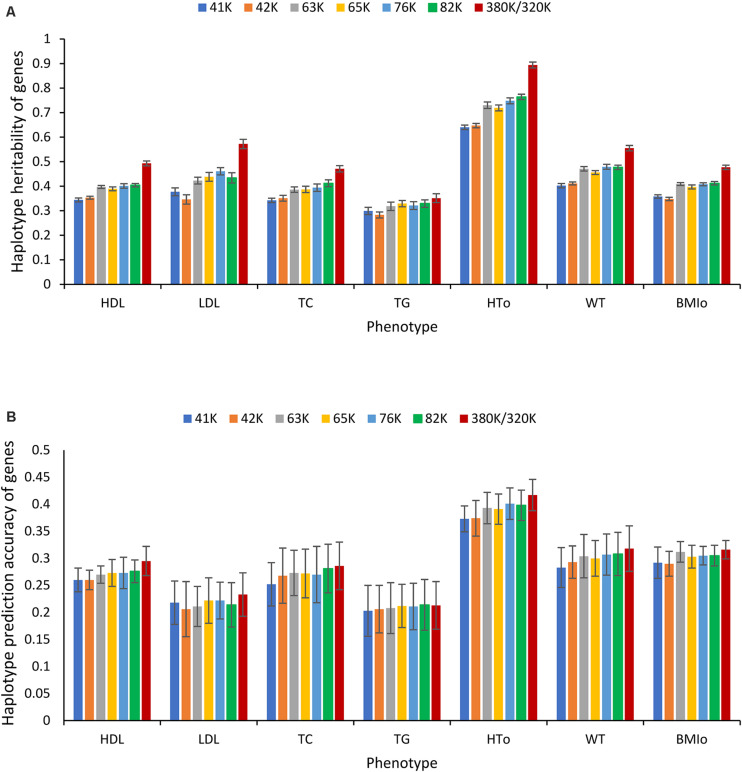
Effect of SNP density on the heritability and prediction accuracy of haplotypes of autosome genes. **(A)** Average haplotype additive heritability of autosome genes from ten-fold validations. **(B)** Average prediction accuracy of haplotype-only model of autosome genes from ten-fold validations. The error bar is one standard deviation above and below the average haplotype heritability or average prediction accuracy, where standard deviation was calculated from the ten-fold validations.

However, for haplotype analysis of autosome genes, the 380K SNP set with the highest density was the best choice. As the SNP density decreased, the number of genes with at least two SNPs for haplotype analysis decreased. For the 380K, the number of autosome genes with at least two SNPs was 18,080, and this number decreased as the SNP density decreased and became 8,609 for the 41K (Supplementary Table 16). Consequently, haplotype heritability and prediction accuracy of genes decreased as the SNP density decreased. The decrease in haplotype heritability due to decreased SNP density (Figure 9A) was more striking than the decrease in haplotype prediction accuracy (Figure 9B). The highest density, the 380K, had the highest prediction accuracy for six of the seven phenotypes with the only exception of TG for which the 320K with 10% MAF was slightly more accurate than the 380K.

### Limitation of This Study

The results of this study have a limitation of being from one study population of Caucasians, the Framingham Heart Study population. It is unknown whether the results from this study also apply to other human populations. However, the consistency between genes with high haplotype and SNP heritabilities and the widely reported significant effects from genome-wide association studies (GWAS) is encouraging that results of this studies may apply to some other populations. For examples, the *CETP*, *APOB*, *CELSR2*, *LPL*, *AC100802.3* genes with high haplotype heritabilties, and *ZBTB38* and *FTO* genes with high SNP heritabilities all had highly significant effects on the same phenotypes from multiple GWAS reports as documented in the GWAS catalog^4^.

## Conclusion

Results in this study showed haplotypes using structural and functional genomic information improved the accuracy of genomic prediction. Haplotypes using structural genomic information covering all autosomes had the best prediction models for most phenotypes, whereas haplotypes using autosome gene boundaries had the best prediction model for one phenotype and tied for the best for another phenotype even though the gene haplotypes only covered 50.78% of the autosomes. Haplotypes using coding gene boundaries covering 37.66% of the autosomes were nearly as accurate as haplotypes based on all autosome genes for two phenotypes and was more accurate than haplotypes based on all autosome genes for two phenotypes. Although ChIP-seq alone did not have the best prediction model for any trait, ChIP-seq had higher prediction accuracy than all autosome genes for one trait, and improved the prediction accuracy over the best SNP models when combined with SNP additive an dominance values for another five traits. These results showed that functionality of the genome is relevant to genomic prediction and that multi-allelic haplotype analysis can be a method to utilize both functional and structural genomic information for genomic prediction. Haplotype epistasis was the reason for the increased prediction accuracy of haplotype models over SNP models. Noncoding genes (mostly lncRNAs) had the highest haplotype epistasis heritability relative to the SNP heritability in the same regions, followed by coding genes and ChIP-seq. These results provided new understanding of the genetic mechanism underlying the accuracy of genomic prediction, and indicated the widespread presence of local epistasis within haplotype blocks affecting the seven human phenotypes, particularly low density lipoproteins that had the largest haplotype epistasis heritability and relative increase in prediction accuracy. The integration of SNP and haplotype additive values in the prediction model improved the prediction for phenotypes where haplotype additive heritability estimates did not identify chromosome locations with high SNP additive heritability estimates, but haplotype-only models were the best prediction models for three phenotypes, indicating that haplotype effects fully accounted for SNP effects or estimated SNP effects more accurately than the SNP models.

## Data Availability Statement

The datasets presented in this study are available from dbGaP, and data access requires approval from the National Heart, Lung, and Blood Institute (NHLBI).

## Author Contributions

YD conceived the study. ZL, CT, DP, and YD analyzed the data. LM processed an early version of the FHS data that were used for preliminary analysis. DP and LM contributed computing tools. YD, ZL, CT, and DP prepared the manuscript. All authors contributed to the article and approved the submitted version.

## Conflict of Interest

The authors declare that the research was conducted in the absence of any commercial or financial relationships that could be construed as a potential conflict of interest.

## Footnotes

1 ftp://ftp.ensembl.org/pub/release-99/variation/vcf/homo_sapiens/

2 ftp://ftp.ensembl.org/pub/release-99/gtf/homo_sapiens/Homo_sapiens.GRCh38.99.gtf.gz

3 http://remap.univ-amu.fr/download_page

4 https://www.ebi.ac.uk/gwas/
