# Supplementary material for: Haplotype Analysis of Genomic Prediction Using Structural and Functional Genomic Information for Seven Human Phenotypes
Source: Front Genet. 2020 Nov 26;11:588907. doi: 10.3389/fgene.2020.588907 (PMC7726221; doi:10.3389/fgene.2020.588907)
Supplement: Supplementary file 1 [file Data_Sheet_1.pdf]

Supplementary Material:

Haplotype analysis of genomic prediction using structural and functional genomic information for seven human phenotypes

Zuoxiang Liang[1,4], Cheng Tan[1,2,4], Dzianis Prakapenka[1], Li Ma[3], Yang Da[1,*]
[1] Department of Animal Science, University of Minnesota, Saint Paul, MN, USA; [2] National Engineering Research Center for Breeding Swine Industry, South China Agricultural University, Guangdong, 510642, China; [3] Department of Animal and Avian Sciences, University of Maryland, College Park, MD, USA;
[4] Contributed equally; [*] Correspondence.

FIGURE S1 | Triglyceride tests in the FHS data by test year.
FIGURE S2 | Distribution of original and normality transformed phenotypic values using Box-Cox transformation of seven human phenotypes. HDL and TC each removed one outlier, and TG removed two outliers.
FIGURE S3 | Optimal λ values for Box-Cox transformation of seven human phenotypes in the FHS data.
FIGURE S4 | Prediction accuracy of haplotype models using fixed chromosome distances and gene boundaries as haplotype blocks from the 380K SNP set.
FIGURE S5 | Prediction accuracy of haplotype models using fixed number of SNPs and gene boundaries as haplotype blocks from the 380K SNP set.
FIGURE S6 | Prediction accuracy of haplotype models using fixed chromosome distances and gene boundaries as haplotype blocks from the 320K SNP set.
FIGURE S7 | Prediction accuracy of haplotype models using fixed number of SNPs and gene boundaries as haplotype blocks from the 320K SNP set.
TABLE S1 | Densities of eight SNP sets for the analysis of haplotype prediction accuracy.
TABLE S2 | Statistics of original and Box-Cox transformed phenotypic values.
TABLE S3 | Statistics of triglycerides in the 2019 version of Framingham Heart Study (FHS) data.
TABLE S4 | SNP prediction accuracy with and without Box-Cox transformation from a 10-fold validation study per trait using the SNP prediction models.
TABLE S5 | Statistics of haplotype blocks defined by fixed chromosome distance (320K, MAF = 0.10).
TABLE S6 | Statistics of haplotype blocks defined by fixed number of SNPs (320K, MAF = 0.10).
TABLE S7 | Noncoding gene types and number of noncoding genes with at least two SNPs per gene for haplotype analysis.
TABLE S8 | Best prediction models and haplotype blocking methods of different SNP densities.
TABLE S9 | Prediction accuracy of different SNP densities from the best prediction models.
TABLE S10 | SNP additive heritability of different SNP densities.
TABLE S11 | SNP dominance heritability of different SNP densities.
TABLE S12 | SNP total heritability as sum of SNP additive and dominance heritabilities of different SNP densities.
TABLE S13 | Total heritability as sum of haplotype additive heritability and SNP additive and dominance heritabilities of different SNP densities from the best prediction models.
TABLE S14 | Haplotype heritability of different SNP densities from the best prediction models.
TABLE S15 | Haplotype epistasis heritability of different SNP densities from the best prediction models.
TABLE S16 | Statistics of haplotype blocks defined by gene boundaries for eight SNP densities.
Text 1: The Variance-based Method (VBM) for Estimating Haplotype Epistasis Heritability

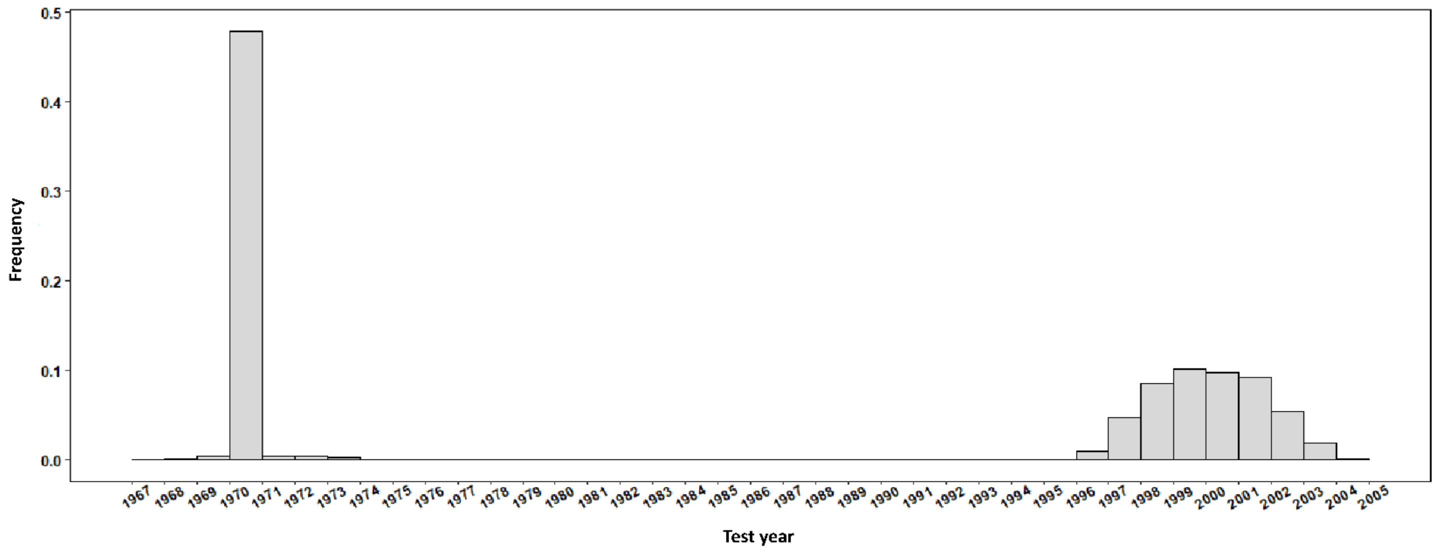

FIGURE S1 | Triglyceride tests in the FHS data by test year.

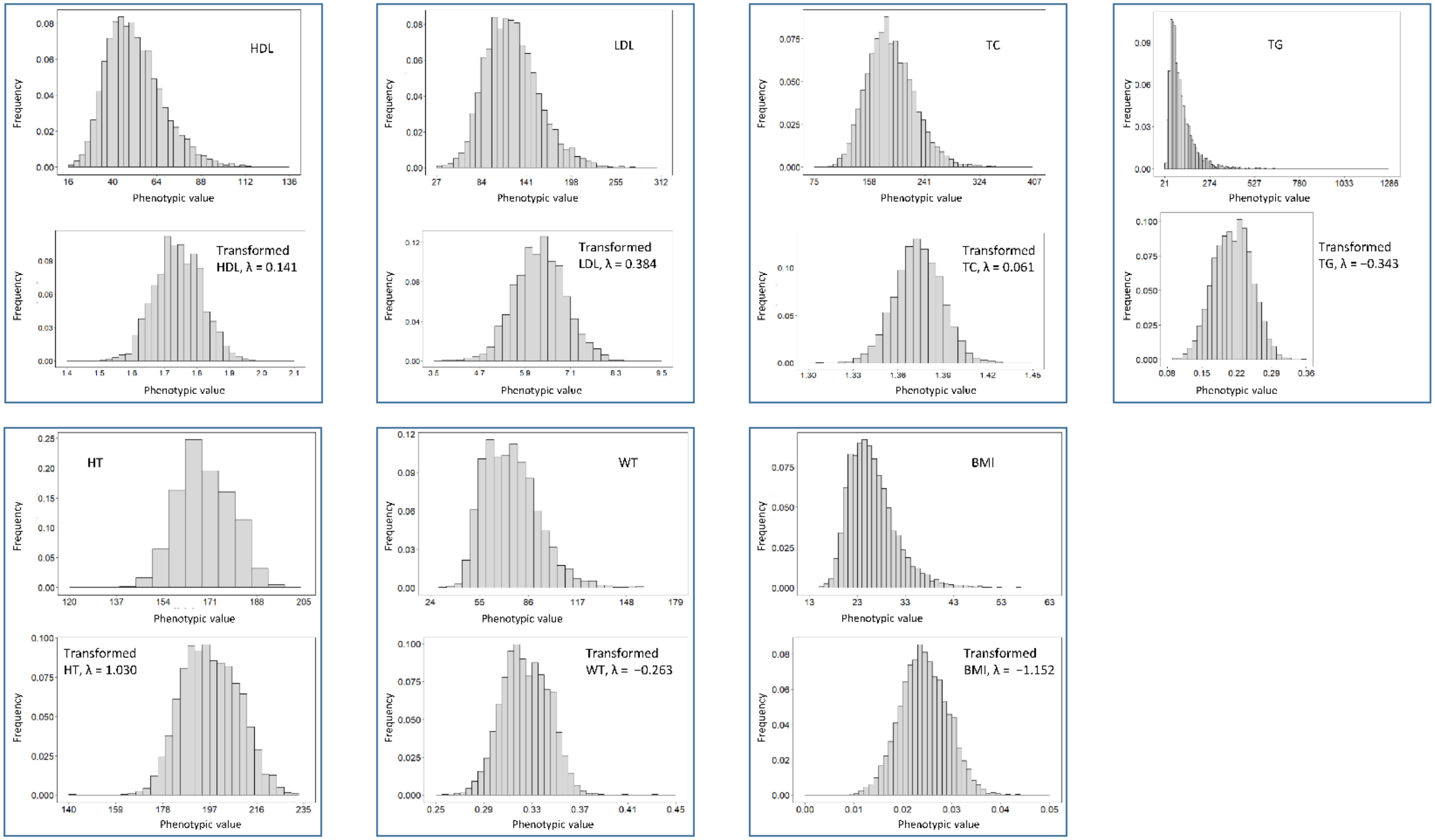

FIGURE S2 | Distribution of original and normality transformed phenotypic values using Box-Cox transformation of seven human phenotypes. HDL and TC each removed one outlier, and and TG removed two outliers.

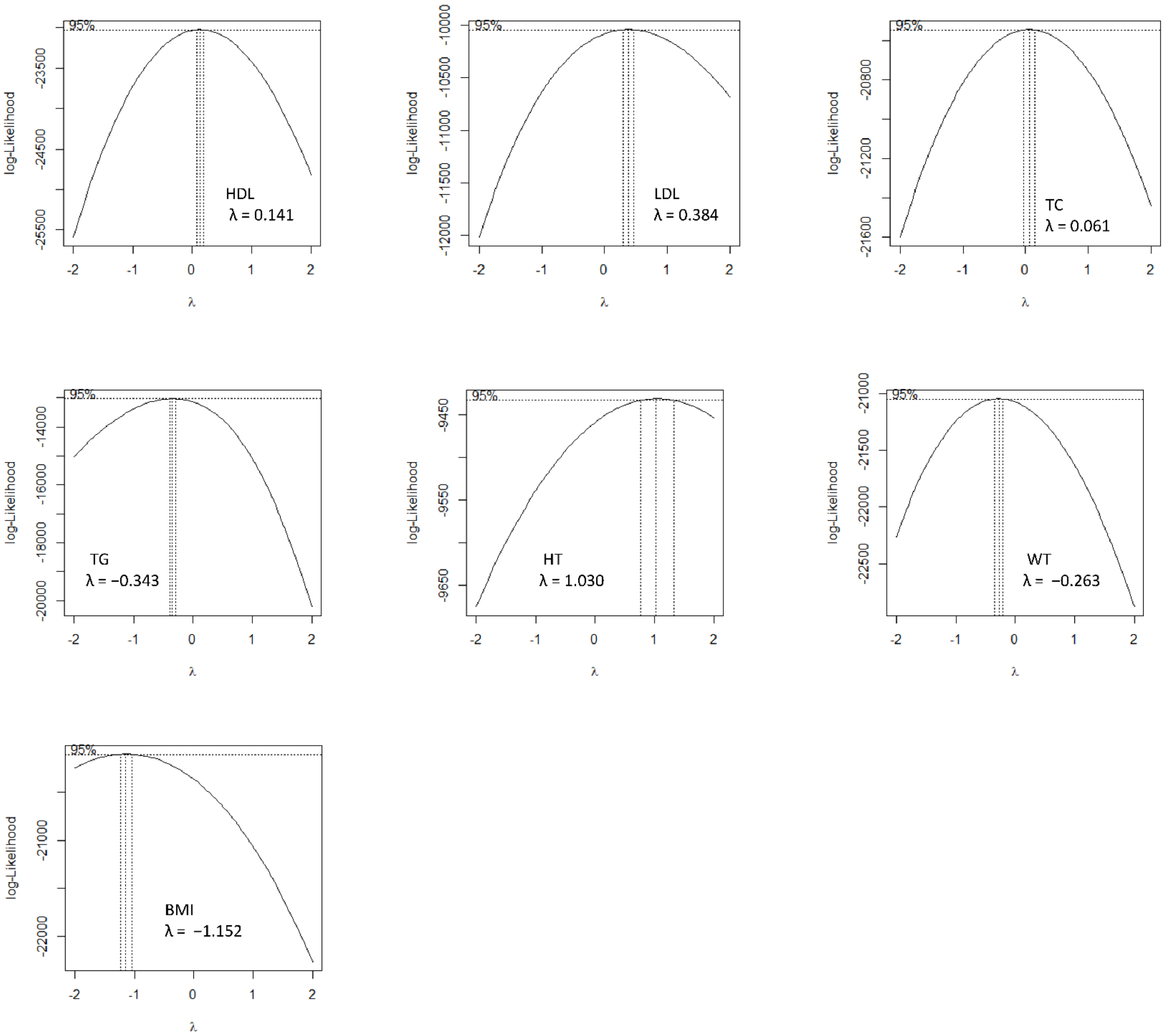

FIGURE S3 | Optimal λ values for Box-Cox transformation of seven human phenotypes in the FHS data.

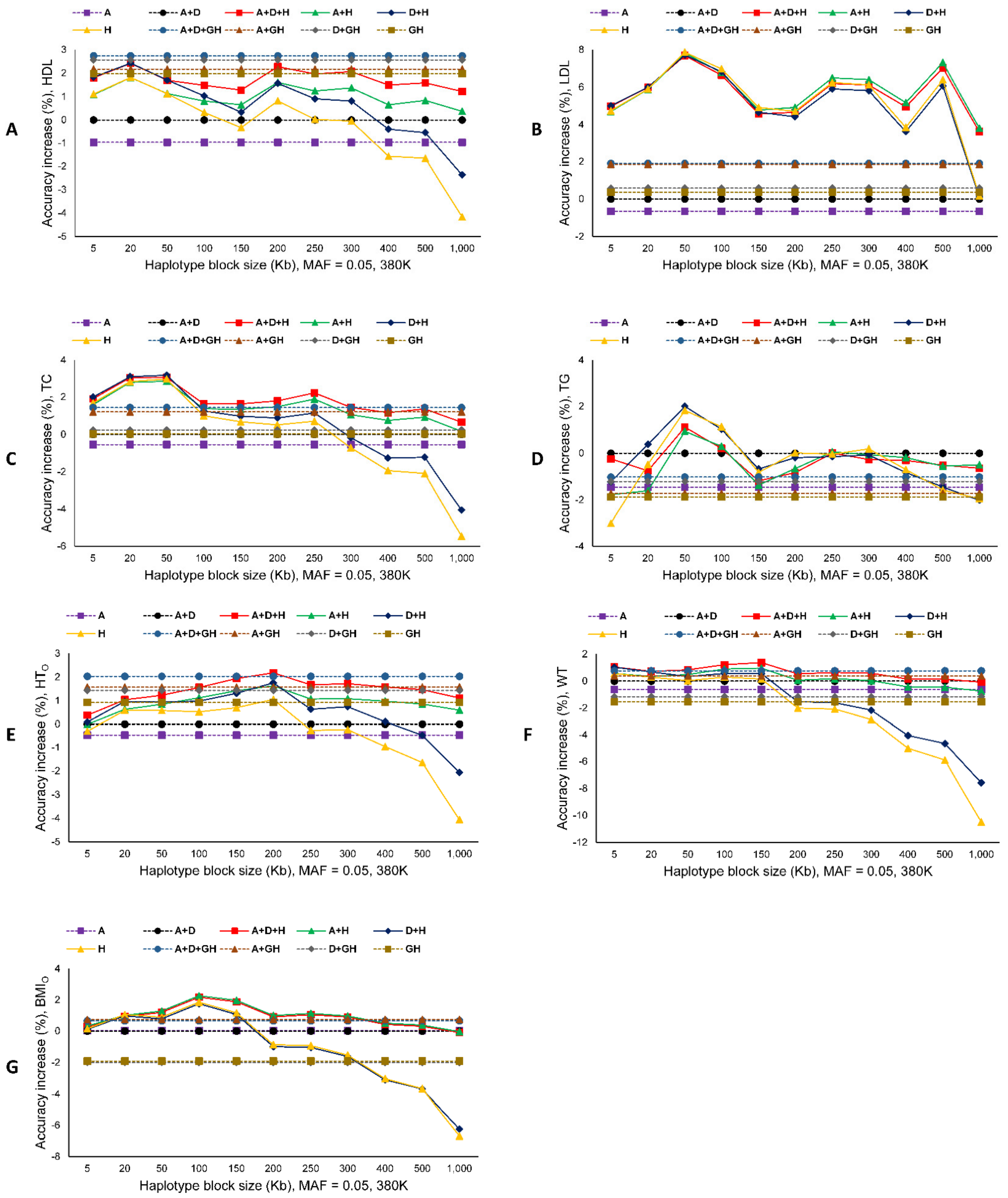

FIGURE S4 | Prediction accuracy of haplotype models using fixed chromosome distances and gene boundaries as haplotype blocks from the 380K SNP set. A = SNP additive value. D = SNP dominance value. H = haplotype additive value. GH = haplotype additive value of a gene.

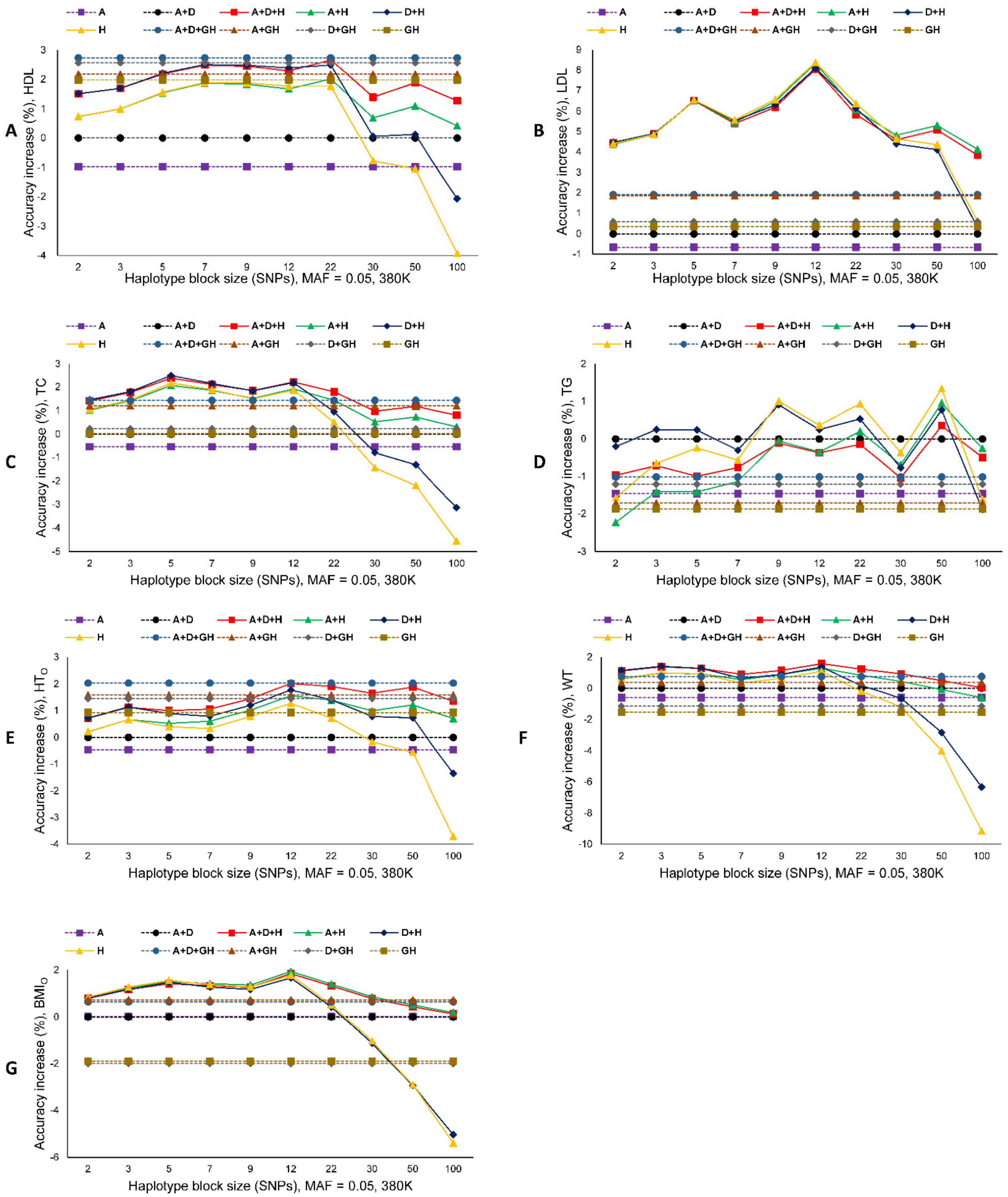

FIGURE S5 | Prediction accuracy of haplotype models using fixed number of SNPs and gene boundaries as haplotype blocks from the 380K SNP set. A = SNP additive value. D = SNP dominance value. H = haplotype additive value. GH = haplotype additive value of a gene.

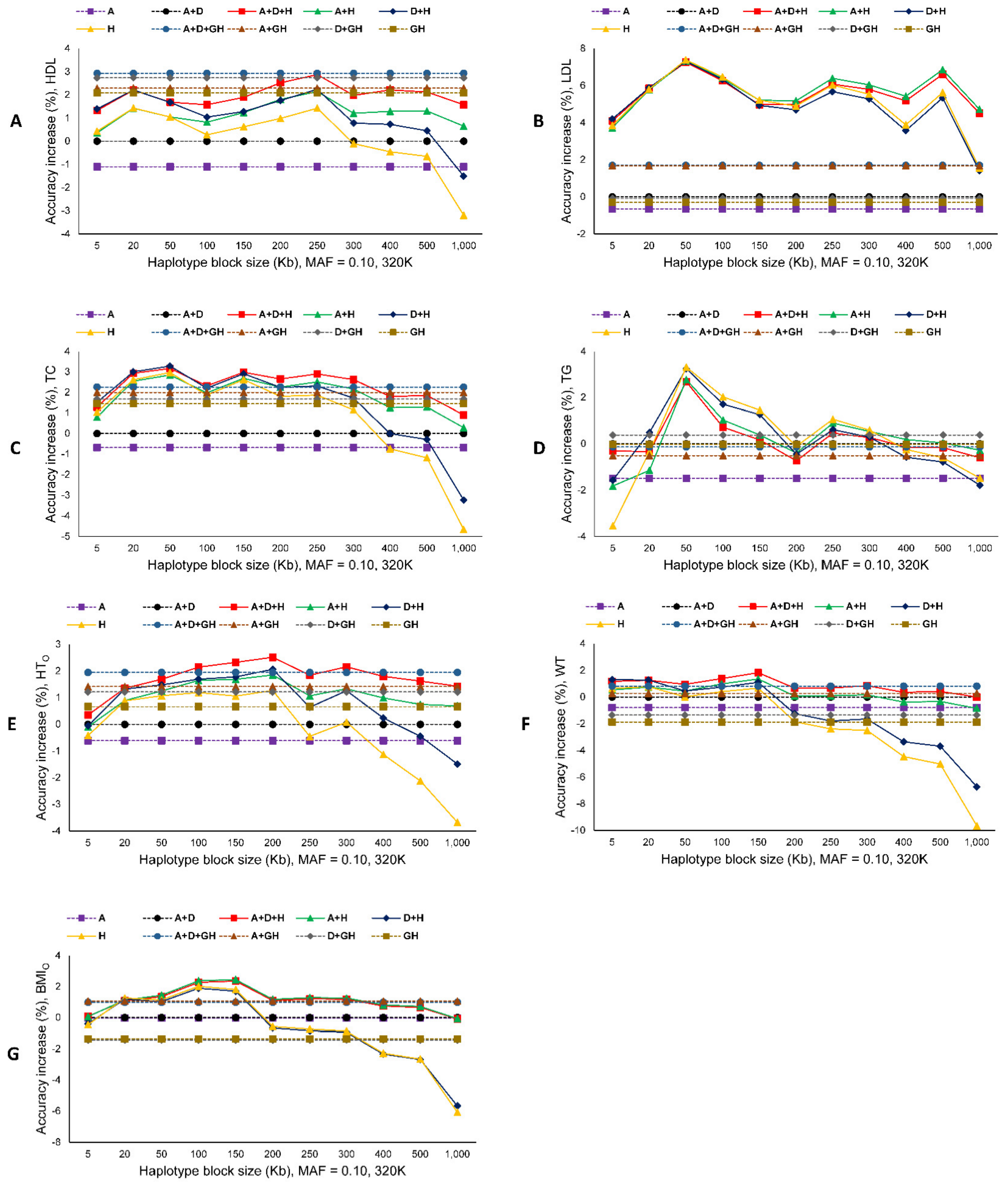

FIGURE S6 | Prediction accuracy of haplotype models using fixed chromosome distances and gene boundaries as haplotype blocks from the 320K SNP set. A = SNP additive value. D = SNP dominance value. H = haplotype additive value. GH = haplotype additive value of a gene.

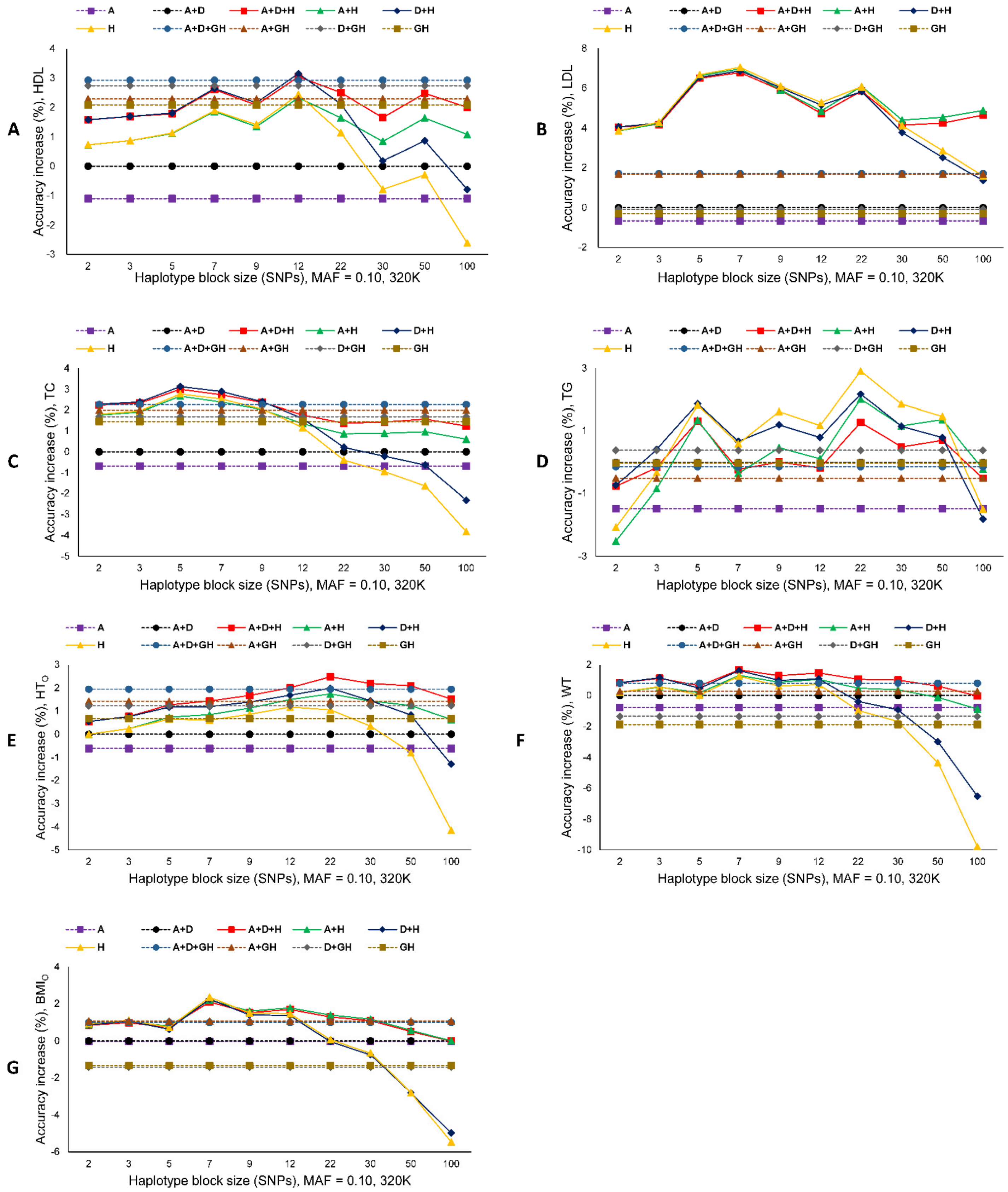

FIGURE S7 | Prediction accuracy of haplotype models using fixed number of SNPs and gene boundaries as haplotype blocks from the 320K SNP set. A = SNP additive value. D = SNP dominance value. H = haplotype additive value. GH = haplotype additive value of a gene.

TABLE S1 | Densities of eight SNP sets for the analysis of haplotype prediction accuracy.

SNP set	MAF	# of SNPs	SNP selection
380K	0.05	380,705	From 486,356 SNPs with MAF=0.05
320K	0.10	327,430	From 486,356 SNPs with MAF=0.10
42K	0.05	42,312	Every 9th SNP of 380K
63K	0.05	63,457	Every 6th SNP of 380K
76K	0.05	76,151	Every 5th SNP of 380K
41K	0.10	40,941	Every 8th SNP of 320K
65K	0.10	65,495	Every 5th SNP of 320K
82K	0.10	81,866	Every 4th SNP of 320K

MAF = minor allele frequency.

TABLE S2 | Statistics of original and Box-Cox transformed phenotypic values.

Trait	λ for Box-Cox transformation	N	Mean	SD	Max	Min
			**Original phenotypic values**			
HDL	–	7491	52.616	15.374	136	16
LDL	–	3657	123.404	34.318	312	29
TG (1996-2005)	–	3835	115.288	86.12	1282	21
TC	–	7508	191.428	36.652	407	76
HT (cm)	–	7564	169.006	9.615	200.025	121.92
WT (kg)	–	7561	75.057	17.584	177.514	24.062
BMI	–	7561	26.115	5.033	60.58	13.528
			**Box-Cox transformed phenotypic values**			
HDL	0.141	7491	1.743	0.071	2.003	1.48
LDL	0.384	3657	6.291	0.677	9.065	3.642
TG (1996-2005)	-0.343	3835	0.211	0.038	0.351	0.086
TC	0.061	7508	1.374	0.016	1.439	1.3
HT (cm)	1.030	7564	197.441	11.572	234.863	141.023
WT (kg)	-0.263	7561	0.325	0.019	0.434	0.257
BMI	-1.152	7561	0.024	0.005	0.05	0.009

HDL= high density lipoproteins (removed one outlier). LDL = low density lipoproteins. TC = total cholesterol (removed one outlier). TG = triglycerides (removed two outliers). HT = height. WT = weight. BMI = body mass index = Weight/(Height/100)^2. HT had $\lambda = 1.03 \approx 1.00$ and hence did not require normality transformation.

TABLE S3 | Statistics of triglycerides in the 2019 version of Framingham Heart Study (FHS) data.

Test period	N	Mean	SD	Max	Min
1967-1974	3670	310.59	285.04	7750	15
1996-2005	3837	115.99	91.35	1499	21

TABLE S4 | SNP prediction accuracy with and without Box-Cox transformation from a 10-fold validation study per trait using the SNP prediction models.

Trait	HDL	LDL	TG	TC	HT	WT	BMI
	**Original phenotypic values, MAF = 0.05**						
Additive only (A)	0.272	0.218	0.156	0.273	0.411	0.319	0.322
Additive and dominance (A+D)	0.274	0.223	0.157	0.273	0.413	0.321	0.322
	**Box-Cox Transformed phenotypic values, MAF = 0.05**						
Additive only (A)	0.285	0.232	0.202	0.295	−	0.322	0.310
Additive and dominance (A+D)	0.289	0.234	0.204	0.295	−	0.323	0.310
	**Original phenotypic values, MAF = 0.10**						
Additive only (A)	0.269	0.217	0.157	0.271	0.408	0.315	0.320
Additive and dominance (A+D)	0.272	0.222	0.158	0.272	0.411	0.319	0.320
	**Box-Cox Transformed phenotypic values, MAF = 0.10**						
Additive only (A)	0.282	0.232	0.202	0.293	−	0.318	0.308
Additive and dominance (A+D)	0.287	0.233	0.204	0.293	−	0.321	0.308

TABLE S5 | Statistics of haplotype blocks defined by fixed chromosome distance (320K, MAF = 0.10).

Distance (Kb)	5	20	50	100	150	200	250	300	400	500	1,000
Total number of haplotypes	909,288	1,940,274	3,962,451	8,051,392	12,302,985	16,065,698	19,080,760	21,402,134	24,163,205	25,262,902	22,541,999
Number of blocks	77,696	75,226	45,691	25,309	17,288	13,111	10,544	8,810	6,641	5,327	2,688
Average number of haplotypes per block	11.7	25.79	86.72	318.12	711.65	1,225.36	1,809.63	2,429.3	3,638.49	4,742.43	8,386.16
Minimum SNPs per block	2	2	2	2	2	2	2	2	2	2	2
Maximum SNPs per block	15	25	43	70	87	106	136	158	207	242	400
Average number of SNPs per block	2.65	3.98	7.08	12.91	18.93	24.97	31.05	37.16	49.3	61.46	121.81
Minimum distance in block (Kb)	5	20	50	100	150	200	250	300	400	500	1,000
Maximum distance in block (Kb)	5	20	50	100	150	200	250	300	400	500	1,000
Average distance per block (Kb)	5	20	50	100	150	200	250	300	400	500	1,000

TABLE S6 | Statistics of haplotype blocks defined by fixed number of SNPs (320K, MAF = 0.10).

Number of SNPs per block	2	3	5	7	9	12	22	30	50	100
Total number of haplotypes	1,273,976	1,539,167	2,351,835	3,364,695	4,511,065	6,422,830	13,383,596	18,328,665	25,857,916	26,574,450
Number of blocks	163,722	109,149	65,495	46,783	36,392	27,294	14,893	10,926	6,560	3,288
Average number of haplotypes per block	7.78	14.1	35.91	71.92	123.96	235.32	898.65	1,677.53	3,941.76	8,082.25
Minimum SNPs per block	2	3	5	7	9	12	22	30	50	100
Maximum SNPs per block	2	3	5	7	9	12	22	30	50	100
Average number of SNPs per block	2	3	5	7	9	12	22	30	50	100
Minimum distance in block (Kb)	0.01	0.03	0.16	0.49	1.24	2.46	15.27	18.9	65.03	187.19
Maximum distance in block (Kb)	21,877.05	22,897.19	23,958.2	29,660.79	24,209.57	29,687.84	29,819.19	29,868.19	29,950.01	30,211.81
Average distance per block (Kb)	8.58	17.11	33.62	51.47	68.27	93.85	178.9	247.17	417.9	843.99

TABLE S7 | Noncoding gene types and number of noncoding genes with at least two SNPs per gene for haplotype analysis.

Type of noncoding gene	Number of genes with at least 2 SNPs	
	380K, MAF =0.05	320K, MAF =0.10
lncRNA	6093	5663
transcribed_unprocessed_pseudogene	229	207
processed_pseudogene	179	163
unprocessed_pseudogene	154	132
transcribed_processed_pseudogene	78	69
transcribed_unitary_pseudogene	70	64
TEC	46	41
unitary_pseudogene	21	17
polymorphic_pseudogene	18	15
misc_RNA	4	0
ribozyme	1	0
snRNA	1	1

Noncoding gene classification was based on the Gene Transfer Format (GTF) files (ftp://ftp.ensembl.org/pub/release-99/gtf/homo_sapiens/Homo_sapiens.GRCh38.99.gtf.gz).

TABLE S8 | Best prediction models and haplotype blocking methods of different SNP densities.

Trait	HDL	LDL	TC	TG	HT_O	WT	BMI_O
41K, MAF=0.10	A+D+H (1,500 Kb)	A+D+H (450 Kb)	A+D+H (1,000 Kb)	H (300 Kb)	A+D+H (1,000 Kb)	A+D+H (300 Kb)	A+H (450 Kb)
42K, MAF=0.05	A+H (1,500 Kb)	H (1,000 Kb)	A+D+H (1,000 Kb)	D+H (150 Kb)	A+D+H (1,000 Kb)	A+D+H (250 Kb)	A+D+H (250 Kb)
63K, MAF= 0.05	A+D+H (1,000 Kb)	D+H (250 Kb)	D+H (250 Kb)	H (450 Kb)	A+D+H (400 Kb)	A+D+H (100 Kb)	A+D+H (250 Kb)
65K, MAF=0.10	A+D+H (450 Kb)	H (350 Kb)	D+H (250 Kb)	D+H (150 Kb)	A+D+H (1,000 Kb)	A+D+H (200 Kb)	A+H (150 Kb)
76K, MAF=0.05	A+D+H (450 Kb)	H (350 Kb)	A+D+H (250 Kb)	H (400 Kb)	A+D+H (1,000 Kb)	A+D+H (250 Kb)	A+H (400 Kb)
82K, MAF=0.10	A+D+H (250 Kb)	H (350 Kb)	D+H (200 Kb)	H (150 Kb)	A+D+H (1,000 Kb)	A+D+H (250 Kb)	A+D+H (300 Kb)
320K, MAF=0.10	D+H (12 SNPs)	H (50 Kb)	D+H (50 Kb)	H (50 Kb)	A+D+H (200 Kb)	A+D+H (150 Kb)	A+H (150 Kb)
380K, MAF=0.05	A+D+H (Gene)	H (12 SNPs)	D+H (50 Kb)	H (50 Kb)	A+D+H (200 Kb)	A+D+H (12 SNPs)	A+H (100 Kb)

TABLE S9 | Prediction accuracy of different SNP densities from the best prediction models.

Trait	HDL	LDL	TC	TG	HT_O	WT	BMI_O
380K, MAF = 0.05	0.298±0.024	0.253±0.050	0.295±0.047	0.220 (320K) ±0.045	0.422±0.026	0.329±0.041	0.329±0.017
41K, MAF = 0.10	0.286±0.025	0.252±0.048	0.287±0.047	0.218±0.052	0.406±0.025	0.310±0.043	0.316±0.019
42K, MAF = 0.05	0.286±0.025	0.253±0.042	0.284±0.047	0.219±0.054	0.415±0.024	0.317±0.038	0.316±0.016
63K, MAF = 0.05	0.287±0.025	0.251±0.050	0.292±0.045	0.215±0.050	0.414±0.024	0.323±0.037	0.322±0.016
65K, MAF = 0.10	0.291±0.020	0.251±0.043	0.289±0.043	0.221±0.049	0.413±0.025	0.319±0.041	0.321±0.019
76K, MAF = 0.05	0.291±0.023	0.255±0.043	0.291±0.047	0.222±0.050	0.417±0.023	0.320±0.039	0.319±0.018
82K, MAF = 0.10	0.291±0.023	0.244±0.047	0.291±0.045	0.217±0.050	0.415±0.022	0.322±0.040	0.321±0.017

TABLE S10 | SNP additive heritability of different SNP densities.

Trait	HDL	LDL	TC	TG	HTo	WT	BMIo
380K, MAF = 0.05	0.386	0.406	0.389	0.257 (320K)	0.739	0.474	0.415
41K, MAF = 0.10	0.344	0.349	0.343	0.269	0.641	0.407	0.367
42K, MAF = 0.05	0.347	0.333	0.339	0.237	0.681	0.425	0.361
63K, MAF = 0.05	0.367	0.354	0.359	0.254	0.691	0.438	0.382
65K, MAF = 0.10	0.356	0.386	0.361	0.241	0.682	0.440	0.380
76K, MAF = 0.05	0.370	0.370	0.366	0.278	0.700	0.447	0.386
82K, MAF = 0.10	0.361	0.380	0.374	0.260	0.704	0.451	0.390

TABLE S11 | SNP dominance heritability of different SNP densities.

Trait	HDL	LDL	TC	TG	HTo	WT	BMIo
380K, MAF = 0.05	0.121	0.174	0.102	0.126 (320K)	0.198	0.088	0.044
41K, MAF = 0.10	0.091	0.189	0.105	0.083	0.139	0.066	0.035
42K, MAF = 0.05	0.058	0.169	0.087	0.130	0.109	0.067	0.047
63K, MAF = 0.05	0.074	0.199	0.092	0.067	0.168	0.110	0.074
65K, MAF = 0.10	0.126	0.131	0.088	0.137	0.185	0.076	0.058
76K, MAF = 0.05	0.096	0.181	0.110	0.070	0.157	0.084	0.046
82K, MAF = 0.10	0.107	0.136	0.085	0.097	0.155	0.082	0.054

TABLE S12 | SNP total heritability as sum of SNP additive and dominance heritabilities of different SNP densities.

Trait	HDL	LDL	TC	TG	HTo	WT	BMIo
380K, MAF = 0.05	0.507	0.58	0.491	0.385 (320K)	0.937	0.562	0.459
41K, MAF = 0.10	0.435	0.537	0.448	0.352	0.779	0.473	0.403
42K, MAF = 0.05	0.405	0.501	0.425	0.367	0.79	0.492	0.408
63K, MAF = 0.05	0.44	0.552	0.451	0.322	0.858	0.547	0.456
65K, MAF = 0.10	0.481	0.516	0.449	0.377	0.867	0.517	0.438
76K, MAF = 0.05	0.466	0.551	0.475	0.347	0.857	0.531	0.431
82K, MAF = 0.10	0.468	0.516	0.459	0.357	0.859	0.533	0.444

TABLE S13 | Total heritability as sum of haplotype additive heritability and SNP additive and dominance heritabilities of different SNP densities from the best prediction models.

Trait	HDL	LDL	TC	TG	HT_O	WT	BMI_O
380K, MAF = 0.05	0.560	0.616	0.530	0.353 (320K)	0.999	0.603	0.488
41K, MAF = 0.10	0.553	0.631	0.530	0.346	0.961	0.530	0.453
42K, MAF = 0.05	0.506	0.672	0.519	0.375	0.942	0.540	0.454
63K, MAF = 0.05	0.538	0.656	0.510	0.363	0.963	0.574	0.502
65K, MAF = 0.10	0.551	0.593	0.500	0.392	0.999	0.560	0.442
76K, MAF = 0.05	0.536	0.628	0.525	0.376	0.999	0.576	0.470
82K, MAF = 0.10	0.529	0.598	0.505	0.343	0.999	0.574	0.485

TABLE S14 | Haplotype heritability of different SNP densities from the best prediction models.

Trait	HDL	LDL	TC	TG	HT_O	WT	BMI_O
380K, MAF = 0.05	0.491	0.616	0.469	0.353 (320K)	0.947	0.567	0.497
41K, MAF = 0.10	0.516	0.572	0.470	0.346	0.831	0.489	0.453
42K, MAF = 0.05	0.528	0.672	0.474	0.313	0.853	0.500	0.433
63K, MAF = 0.05	0.515	0.586	0.447	0.363	0.832	0.493	0.457
65K, MAF = 0.10	0.470	0.593	0.438	0.352	0.870	0.513	0.444
76K, MAF = 0.05	0.479	0.628	0.452	0.376	0.907	0.529	0.476
82K, MAF = 0.10	0.461	0.598	0.447	0.343	0.898	0.527	0.463

TABLE S15 | Haplotype epistasis heritability of different SNP densities from the best prediction models.

Trait	HDL	LDL	TC	TG	HT_O	WT	BMI_O
380K, MAF = 0.05	0.053	0.147	0.036	0.083 (320K)	0.062	0.041	0.064
41K, MAF = 0.10	0.118	0.094	0.082	0.046	0.182	0.057	0.079
42K, MAF = 0.05	0.147	0.291	0.094	0.008	0.152	0.048	0.046
63K, MAF = 0.05	0.098	0.104	0.059	0.08	0.105	0.027	0.046
65K, MAF = 0.10	0.07	0.164	0.051	0.015	0.132	0.043	0.049
76K, MAF = 0.05	0.07	0.199	0.05	0.072	0.142	0.045	0.075
82K, MAF = 0.10	0.061	0.176	0.046	0.044	0.14	0.041	0.041

TABLE S16 | Statistics of haplotype blocks defined by gene boundaries for eight SNP densities.

	41K, MAF = 0.10	42K, MAF = 0.05	63K, MAF = 0.05	65K, MAF = 0.10	76K, MAF = 0.05	82K, MAF = 0.10	320K, MAF = 0.10	380K, MAF = 0.05
Total number of haplotypes	163,864	167,505	418,070	481,588	634,036	797,246	6,350,392	7,419,624
Number of blocks	8,609	8,951	11,423	11,407	12,325	12,535	17,238	18,080
Average number of haplotypes per block	19.03	18.71	36.6	42.22	51.44	63.6	368.39	410.38
Minimum SNPs per block	2	2	2	2	2	2	2	2
Maximum SNPs per block	10	10	15	16	18	21	82	87
Average number of SNPs per block	2.91	2.92	3.76	3.9	4.28	4.57	12.11	13.49
Minimum distance in block (Kb)	4.3	4.29	4.07	4.07	4.07	4.07	1.14	1.14
Maximum distance in block (Kb)	150.0	150.0	150.0	150.0	150.0	150.0	150.0	150.0
Average distance per block (Kb)	138.3	138.36	132.75	132.16	130.25	128.52	92.65	90.13
Autosome coverage (Mb)	992.96	1028.62	1250.97	1241.41	1320.30	1325.27	1528.13	1557.23
% of autosomes	30.94	32.05	38.98	38.69	41.14	41.30	47.62	48.53
4-Kb extended coverage (Mb)	1019.72	1056.30	1286.19	1276.61	1358.38	1364.21	1597.08	1629.55
% of autosomes by 4-Kb extended coverage	31.78	32.92	40.08	39.78	42.33	42.51	49.77	50.78

Text 1: The Variance-based Method (VBM) for Estimating Haplotype Epistasis Heritability

Haplotype epistasis heritability is defined as the contribution of haplotype epistasis variance to the phenotypic variance, and can be estimated using two methods, variance-base method (VBM) and heritability-based method (HBM). The VBM method may have numerical problems and the HBM method is used in this study and is described in the main text. The following describes the VBM method for the four haplotype models, the H model, A+H model, D+H model, and A+D+H model, where A = SNP additive values, D = SNP dominance values, and H = haplotype additive values.

Haplotype-only model (H model, Model 4)

Based on the empirical hypothesis that a haplotype additive value is the summation of the SNP additive values and a haplotype epistasis value within the haplotype, plus a potential haplotype loss of SNP effects (Da et al., 2016),

$$h = a + \varepsilon + \tau \qquad \text{(S1)}$$

where h = haplotype additive value, a = additive values of all SNPs in the haplotype, ε = haplotype epistasis value, and τ = haplotype loss. From the model of Equation S1, the haplotype additive variance can be expressed as:

$$\sigma^2_{\alpha h} = \sigma^2_{\alpha} + \sigma^2_{E} + \sigma^2_{\tau} \qquad \text{(S2)}$$

where $\sigma^2_{\alpha h}$ = haplotype additive variance, σ^2_{α} = the unobservable SNP additive variance from the SNP additive values contained in haplotypes, σ^2_{E} = haplotype epistasis variance, and σ^2_{τ} = haplotype loss variance.

In cases where the haplotype-only model is the best prediction model so that adding SNP effects to the prediction model decreases the prediction accuracy, haplotype loss can be assumed nonexistent or negligible, and Equation S2 reduces to:

$$\sigma^2_{\alpha h} = \sigma^2_{\alpha} + \sigma^2_{E} \qquad \text{(S3)}$$

From Equation S3, the haplotype epistasis variance and heritability for the haplotype-only model (Model 4) are:

$$\sigma^2_{E} = \sigma^2_{\alpha h} - \sigma^2_{\alpha 1} \qquad \text{(S4)}$$

$$\hat{h}^2_{EV} = \sigma^2_{E} / (\sigma^2_{\alpha h} + \sigma^2_{e}) \qquad \text{(S5)}$$

where σ^2_{e} = residual variance, $\sigma^2_{\alpha 1}$ = SNP additive variance from the model with SNP additive values only (the A model, Model 6). In this study, the haplotype-only model was the best prediction model for low density lipoproteins (LDL) and triglycerides (TG), and Equations S3-S5 apply to these two traits.

A+H model (Model 2)

Based on the invariance property that GBLUP and GREML are unaffected by duplicate SNPs (Da et al., 2016; Tan et al., 2017), the genotypic value from combining haplotype and SNP additive values predicts only one set of SNP additive values, i.e.,

$$g = a + h \approx a + \varepsilon + \tau \quad \text{(S6)}$$

From Equation S6, the genotypic variance under Model 2 is:

$$\sigma_g^2 = \sigma_{\alpha s}^2 + \sigma_{\alpha h}^2 \approx \sigma_\alpha^2 + \sigma_E^2 + \sigma_\tau^2 \quad \text{(S7)}$$

where $\sigma_{\alpha s}^2$ = SNP additive variance from the A+H model (Model 2), and σ_α^2 = the unobservable SNP additive variance from the duplicated SNP additive values contained in haplotypes and from SNPs. From Equation S7, the haplotype epistasis variance and heritability are:

$$\sigma_E^2 + \sigma_\tau^2 = \sigma_g^2 - \sigma_{\alpha 1}^2 \quad \text{(S8)}$$

$$\hat{h}_{EV}^2 = (\sigma_E^2 + \sigma_\tau^2) / (\sigma_\alpha^2 + \sigma_E^2 + \sigma_\tau^2 + \sigma_e^2) = (\sigma_g^2 - \sigma_{\alpha 1}^2) / (\sigma_{\alpha s}^2 + \sigma_{\alpha h}^2 + \sigma_e^2) \quad \text{(S9)}$$

In this study, the A+H model was the best prediction model for the original body mass index (BMIO) without normality transformation, and Equations S7-S9 apply to these this trait. In Equations S7-S9, σ_E^2 and σ_τ^2 are confounded in the sense the current methods do not have a mechanism to separate these two variances for Model 2, and for Model 1 as described later. Placing σ_τ^2 in both the numerator and denominator minimizes the impact of σ_τ^2 on the estimation of haplotype epistasis heritability. Since the use of the A+H model assumes the H model (haplotype additive only) is less accurate than the A model (SNP additive only), σ_τ^2 should not be assumed nonexistent.

D+H model (Model 3)

For the D+H model, the genotypic value and variance are:

$$g = d + h \approx a + d + \varepsilon + \tau \quad \text{(S10)}$$

$$\sigma_g^2 = \sigma_{\delta s}^2 + \sigma_{\alpha h}^2 \approx \sigma_\alpha^2 + \sigma_{\delta s}^2 + \sigma_E^2 + \sigma_\tau^2 \quad \text{(S11)}$$

where σ_α^2 = the unobservable SNP additive variance from the SNP additive values contained in haplotypes, $\sigma_{\delta s}^2$ = SNP dominance variance from the model with SNP additive and dominance values (the A+D model, Model 5).

The use of the D+H model implies that SNP additive values in the prediction model reduced the prediction accuracy and that haplotype loss nonexistent or negligible. Therefore, Equation S11 simplifies to:

$$\sigma_g^2 = \sigma_{\delta s}^2 + \sigma_{\alpha h}^2 = \sigma_\alpha^2 + \sigma_{\delta s}^2 + \sigma_E^2 \quad \text{(S12)}$$

and the haplotype epistasis variance and heritability are:

$$\sigma_E^2 = \sigma_g^2 - (\sigma_{\alpha 2}^2 + \sigma_\delta^2) \quad \text{(S13)}$$

$$\hat{h}_{EV}^2 = \sigma_E^2 \, / \, (\sigma_{\delta s}^2 + \sigma_{\alpha h}^2 + \sigma_e^2) \quad \text{(S14)}$$

In this study, the D+H model was the best prediction model for total cholesterol (TC), and Equations S10-S14 apply to this trait.

A+D+H model (Model 1)

For the A+D+H model, the genotypic variance and the haplotype epistasis variance and heritability are:

$$\sigma_g^2 = \sigma_{\alpha s}^2 + \sigma_{\delta s}^2 + \sigma_{\alpha h}^2 \approx \sigma_{\alpha s}^2 + \sigma_{\delta s}^2 + \sigma_E^2 + \sigma_\tau^2 \quad \text{(S15)}$$

$$\sigma_E^2 + \sigma_\tau^2 = \sigma_g^2 - (\sigma_{\alpha 2}^2 + \sigma_\delta^2) \quad \text{(S16)}$$

$$\hat{h}_{EV}^2 = (\sigma_E^2 + \sigma_\tau^2) \, / \, (\sigma_\alpha^2 + \sigma_\delta^2 + \sigma_E^2 + \sigma_\tau^2 + \sigma_e^2) = (\sigma_E^2 + \sigma_\tau^2) \, / \, (\sigma_{\alpha s}^2 + \sigma_{\delta s}^2 + \sigma_{\alpha h}^2 + \sigma_e^2) \quad \text{(S17)}$$

In this study, the A+D+H model was the best model for three traits, high density lipoproteins (HDL), original height (HT_O) without normality transformation, and weight (WT).

Relative haplotype epistasis heritability

Relative haplotype epistasis heritability is defined as the ratio of the haplotype epistasis heritability to the SNP additive heritability, as a measure of the size of haplotype epistasis heritability relative to SNP additive heritability. Depending on the prediction model with haplotypes, estimated relative haplotype epistasis heritability is:

$$\hat{h}_{Erv}^2 = \hat{h}_{EV}^2 / \hat{h}_{\alpha 1}^2 \quad \text{for Models 2 and 4} \quad \text{(S18)}$$

$$\hat{h}_{Erv}^2 = \hat{h}_{EV}^2 / \hat{h}_{\alpha 2}^2 \quad \text{for Models 1 and 3} \quad \text{(S19)}$$

Numerical instability of the VBM method for estimating haplotype epistasis heritability

The VBM method has a problem of numerical instability for two phenotypes, HDL and BMI_O. The estimate of haplotype epistasis heritability was 0.396 for HDL (**Table T1**). For the other six phenotypes, the VBM and HBM methods had similar estimates, where the HBM method is described in the main text. The 0.396 estimate was 102.63% of the SNP additive heritability under the A+D model and was unlikely to be true, given that the accuracy increase due to haplotypes for HDL (2.76%) was less than that for LDL (8.12%) that had nearly the same estimates of haplotype epistasis heritability by both the VBM and HBM methods, 0.1463 by VBM and 0.1468 by the HBM.

The reason for the extreme value of 0.396 for HDL was due to the larger variance components under the A+D+H model than under the A+D model, e.g., the residual variance was 2.80 times as large and the genetic variance was 3.44 times as large as those under the A+D model. Consequently, the comparison between the genetic variances of the two models was inflated by the systematically larger estimates of variance components for unknown reasons. In contrast, the HBM method tends to cancel the factor that caused the systematically large estimates of variance components because the genetic variance is the numerator and denominators of the heritability contains both genetic and residual variances. For the seven phenotypes in this study, the HBM method did not have extreme estimates of haplotype epistasis heritability. Other than HDL, the VBM and HBM methods had similar estimates of haplotype epistasis heritability.

The HBM method had slightly higher estimates of haplotype epistasis heritability than those from the VBM method (**Table T1**) and could have an upward bias in estimates of haplotype epistasis heritability. However, the HBM method was used in the main text because the HBM method did not have the problem of numerical instability as observed for the VBM method for HDL.

TABLE T1 | Calculation of haplotype epistasis heritability using VBM and HBM methods. Blank entries were not needed and '− ' indicates 'unavailable' under the model.

Trait	HDL	LDL	TC	TG	HT_O	Weight	BMI_O
	**SNP model with additive values (A)**						
Additive variance ($\sigma^2_{\alpha 1}$)		0.1792		0.0004			9.7225
Residual variance (σ^2_e)		0.2026		0.0008			13.1949
Phenotypic variance (σ^2_y)		0.3819		0.0012			22.9175
Additive heritability ($\hat{h}^2_{s1}=\hat{h}^2_{\alpha 1}$)	0.409	0.469	0.409	0.312	0.773	0.493	0.4242
	**SNP model with additive and dominance values (A+D)**						
Additive variance ($\sigma^2_{\alpha 2}$)	0.0015		0.0001		4.9086	0.0001	
Dominance variance (σ^2_δ)	0.0005		0.0000		1.4367	0.0000	
Residual variance (σ^2_e)	0.0019		0.0001		0.4375	0.0001	
Phenotypic variance (σ^2_y)	0.0038		0.0002		6.7829	0.0002	
Additive heritability ($\hat{h}^2_{\alpha 2}$)	0.386	0.406	0.389	0.260	0.739	0.474	0.415
Dominance heritability ($\hat{h}^2_\delta$)	0.121	0.174	0.102	0.125	0.198	0.088	0.044
SNP total heritability ($\hat{h}^2_s$)	0.507	0.580	0.491	0.385	0.937	0.562	0.459
	**Haplotype prediction models**						
Best prediction model	A+D+H	H	D+H	H	A+D+H	A+D+H	A+H
Additive variance ($\sigma^2_{\alpha s}$)	0.0008	−	−	−	2.3883	0.00002	2.8023
Dominance variance ($\sigma^2_{\delta s}$)	0.0011	−	0.00002	−	0.9750	0.00001	−
Haplotype variance ($\sigma^2_{\alpha h}$)	0.0047	0.2350	0.00010	0.0004	3.2870	0.00007	8.3548
Residual variance (σ^2_e)	0.0052	0.1465	0.00010	0.0008	0.0004	0.00007	11.7008
Phenotypic variance (σ^2_y)	0.0118	0.3815	0.00022	0.0012	6.6507	0.00016	22.8580
SNP additive heritability ($\hat{h}^2_{\alpha s}$)	0.070	−	−	−	0.359	0.124	0.123
SNP dominance heritability ($\hat{h}^2_{\delta s}$)	0.094	−	0.077	−	0.147	0.057	−
Haplotype additive heritability ($\hat{h}^2_{\alpha h}$)	0.394	0.616	0.452	0.353	0.494	0.422	0.366
Total heritability ($\hat{h}^2_g$)	0.583	0.616	0.530	0.353	0.999	0.603	0.488
	**Haplotype epistasis heritability**						
$\hat{h}^2_{EV}$, VBM method	**0.3960**	0.1463	0.03866	0.0450	0.0459	0.03863	0.0628
$\hat{h}^2_E$, HBM method	0.0512	0.1468	0.03887	0.0448	0.0644	0.04222	0.0639
	**Relative haplotype epistasis heritability**						
$\hat{h}^2_{Erv}$ (%), VBM method	**102.63**	31.16	9.94	14.59	6.34	8.15	14.79
$\hat{h}^2_{Er}$ (%), HBM method	13.27	31.27	9.99	14.53	8.90	8.91	15.05
